# Nodal-induced L1CAM/CXCR4 subpopulation sustains tumor growth and metastasis in colorectal cancer derived organoids

**DOI:** 10.7150/thno.54027

**Published:** 2021-03-31

**Authors:** Donatella Delle Cave, Xavier Hernando-Momblona, Marta Sevillano, Gabriella Minchiotti, Enza Lonardo

**Affiliations:** 1 Institute of Genetics and Biophysics'Adriano Buzzati-Traverso' (IGB), CNR, Via Pietro Castellino 111, 80131 Naples, Italy.; 2 Institute for Research in Biomedicine Barcelona (IRB), Barcelona, Spain.

**Keywords:** colorectal cancer (CRC), organoids, transforming growth factor (TGF)-β signaling, L1 cell adhesion molecule (L1CAM, CD171)

## Abstract

**Background:** Colorectal cancer (CRC) is currently the third leading cause for cancer-related mortality. Cancer stem cells have been implicated in colorectal tumor growth, but their specific role in tumor biology, including metastasis, is still uncertain.

**Methods:** Increased expression of L1CAM, CXCR4 and NODAL was identified in tumor section of patients with CRC and in patients-derived-organoids (PDOs). The expression of L1CAM, CXCR4 and NODAL was evaluated using quantitative real-time PCR, western blotting, immunofluorescence, immunohistochemistry and flow cytometry. The effects of the L1CAM, CXCR4 and NODAL on tumor growth, proliferation, migration, invasion, colony-formation ability, metastasis and chemoresistance were investigated both *in vitro* and *in vivo*.

**Results:** We found that human colorectal cancer tissue contains cancer stem cells defined by L1CAM^high^/CXCR4^high^ expression that is activated by Nodal in hypoxic microenvironment. This L1CAM^high^/CXCR4^high^ population is tumorigenic, highly resistant to standard chemotherapy, and determines the metastatic phenotype of the individual tumor. Depletion of the L1CAM^high^/CXCR4^high^ population drastically reduces the tumorigenic potential and the metastatic phenotype of colorectal tumors.

**Conclusion:** In conclusion, we demonstrated that a subpopulation of migrating L1CAM^high^/CXCR4^high^ is essential for tumor progression. Together, these findings suggest that strategies aimed at modulating the Nodal signaling could have important clinical applications to inhibit colorectal cancer-derived metastasis.

## Introduction

Colorectal cancer (CRC) is currently the third leading cause for cancer-related mortality 1. Despite growing comprehension in tumor biology, the treatment efficiency in CRCs has not improved significantly over the past decade. Several studies on CRC have been focused on the role of cancer stem cells (CSCs). Indeed, cells bearing stem cell properties are involved in self-renewal, tumor progression, apoptosis resistance and cancer relapse following treatment 2,3.

Nodal belongs to transforming growth factor (TGF)-β super family of growth and differentiation factors expressed in various tissues. Through binding with its receptors, Nodal exerts its biological effects by activating the intracellular signaling pathway. Nodal receptors are trans-membrane serine/threonine kinases, including type I (ActRI, named ALK4 and ALK7) and type II (ActRII) receptor. ActRIs combines with ActRIIs to form a complex to transduce Nodal signals. After Nodal binding, ActRI is capable to phosphorylate Smad2 and Smad3. Phosphorylated Smad2/3, together with Smad4, translocate to the nucleus where they regulate the transcription of NODAL target genes 4,5. Interestingly, Nodal pathway may determine the tumor cell progression and metastatic ability by regulating cancer stem cells self-renewal in CRC 6. Besides, Nodal is activated in hypoxic tumor microenvironment 7. Hypoxia enables a number of events in the tumor microenvironment that lead to the expansion of aggressive clones from heterogeneous tumor cells thus promoting a lethal phenotype 8.

Overexpression of L1 cell adhesion molecule (L1CAM; CD171) has been reported in numerous human cancers, including breast, kidney and lung 9. In colorectal cancer cells, L1CAM promotes cell growth and survival and L1CAM secreted from tumor cells makes these cells more invasive and mobile and thus, more aggressive 10. Conversely, L1CAM has an anti-oncogenic function in pancreatic cancer 11.

In CRC it has been demonstrated that abnormal expression of Chemokine (C-X-C motif) receptor 4 (CXCR4) plays a crucial role in the invasion and liver metastasis 12. CXCR4 belongs to G protein-coupled receptor superfamily, which selectively binds to stromal cell-derived factor 1 (SDF-1, also called CXCL12) to promote cancer metastasis 13. Corroborating this data, high expression of CXCR4 correlates with poor prognosis in CRC patients 14,15.

Here, we identified a subpopulation of L1CAM^high^/CXCR4^high^ CSCs activated by Nodal signaling in hypoxic microenvironment that is not only endowed with tumor-initiating properties, but is also capable of forming liver metastasis.

## Methods

### Bioinformatics analysis

Survival was analyzed using the http://genomics.jefferson.edu/proggene/filter.php. A Median Group cut-off (50% high vs 50% Low) was used for L1CAM and (25% high vs 75% Low) was used for CXCR4. GSE40967 contains gene expression profile data of 566 colon cancer patients. *NODAL* transcript expression in colon tumor (T, n = 272) and normal epithelium (N, n = 41) was analyzed using the http://gepia2.cancer-pku.cn/#survival.

### Culture of patient-derived tumor organoids

Human biological samples used to expand organoids were obtained from individuals treated at Hospital del Mar and Hospital de la Santa Creui Sant Pau, under informed consent and approval of the Tumor Bank Committees according to Spanish ethical regulations. The study followed the guidelines of the Declaration of Helsinki and patients identity of pathological specimens remained anonymous in the context of this study. MTA 27/04/2017 n.324. CRC cells with high EphB2 levels were FACS sorted from dissociated tumors and cultured embedded in Matrigel (Basement Membrane Matrix Low Concentration, BD) with Advanced DMEM/F12, 10 mM HEPES, 1× Glutamax; 1× B-27 without retinoic acid, 1× N-2, 20 ng/mL bFGF (basic fibroblast growth factor); 50 ng/mL EGF (epidermal growth factor), 1 μM LY2157299 and 10 μM Y-27632. Under these conditions, cells with high EphB2 levels expanded as tumor organoids that we could propagate indefinitely, whereas cells with medium or low EphB2 levels did not. All cells were tested weekly for mycoplasma contamination with negative results.

### Cell cultures

The human colon cancer cell lines SW480 (tissue derivation: primary colon tumor; carcinoma type: adenocarcinoma) and SW620 (tissue derivation: colon, derived from metastatic site: lymph node; carcinoma type: adenocarcinoma) were cultured in Dulbecco's Modified Eagle Medium (DMEM), supplemented with 10% fetal bovine serum (FBS) and 50 U/mL penicillin/streptomycin at 37 °C in a 5% CO_2_ atmosphere. Their Mycoplasma free-state was tested by using the PCR-based MycoAlert Mycoplasma Detection Kit (Lonza, Bioscience). Each cell line was used for passage 4/5 after thawing from originally frozen vials.

### Immunohistochemistry

Immunostainings were carried out using 4 μm tissue sections according to standard procedures. Briefly, after antigen retrieval, samples were blocked with Peroxidase-Blocking Solution (Dako, S202386) for 10 min at RT, and then primary antibodies were incubated overnight. Slides were washed with EnVision™ FLEX Wash Buffer (Dako, K800721) and the corresponding secondary antibody was incubated with the sample for 45 min at RT. Samples were developed using 3,3′-diaminobenzidine, counterstained with hematoxylin and mounted. See Antibodies section. Images were acquired using a digital image scanning (Nanozoomer 2.0HT, Hamamatsu) and cropped using NDP.view2. The antibodies used are listed in Supplementary **Table S1.**

### Immunofluorescence

L1CAM-PE, Ki67-FITC, CXCR4-FITC, E-CADHERIN-FITC antibody and Phalloidin-TRITC (see Antibodies section) were incubated for 30 min at room temperature in the dark. Unconjugated pSMAD2, NODAL and YAP were incubated over night at 4 °C and the day after counterstained with fluorescent secondary antibody. Cell nuclei were stained with DAPI and slides were mounted in Glycerol/PBS/Phenylenediamine for observation using an SP5 or SPE confocal microscope (Leica). The antibodies used are listed in Supplementary **Table S1.**

### Quantitative RT-PCR (qRT-PCR)

RNA was extracted using Trizol Reagent (Invitrogen) and qRT-PCR was performed using TaqMan assays (Applied Biosystems) or SYBR Green (Applied Biosystems) following manufacturer's instructions. The primers and probes used are listed in Supplementary **Table S2 and S3.**

### Flow cytometry and cell sorting

To identify CSCs anti-human membranous L1CAM-PE and anti-human CXCR4-FITC were used. 7AAD (BD) was used for exclusion of dead cells. Samples (n > 6) were run on the FACSAriaIII Cell Sorter (BD) and data were analysed using FlowJo 9.2 (Ashland, OR). The antibodies used are listed in Supplementary **Table S1.**

### Protein isolation and western blot analysis

Cells were lysed with RIPA buffer (50mM Tris-HCl at pH 7.6, 150 mM NaCl, 1% NP-40, 0.5% sodium deoxycholate, 0.1% SDS, 5 mM EDTA plus proteases and phosphatases inhibitors) for 1hr at 4 °C. Total protein quantification was performed with Bio-Rad Protein Assay Dye Reagent concentrate. A total of 50 μg of protein was separated on 15% SDS-PAGE gels at 100 V and transferred to PVDF membranes for 2 h at 200 mA. PVDF membranes were hybridized with mouse antibodies against L1CAM (HPA005830, Sigma-Aldrich), HIF1a (2015-1, Epitomics), GAPDH (ab9483, Abcam), NODAL (ab55676, Abcam), CXCR4 (ab124824, Abcam), pSmad2 (3108; Cell Signaling) and Smad2 (#5339; Cell Signaling), treated with peroxidase-conjugated goat anti-mouse or anti-rabbit Ig secondary antibody (DPVR-HRP, Immunologic), and then visualized by enhanced chemiluminescence (ECL Nova 2.0 XLS071, 2050 Cyanagen). N > 6. The antibodies used are listed in Supplementary **Table S1.**

### Lentiviral shRNA delivery

As lentiviral shuttle backbone we used a pLKO shRNA plasmid (Mission SIGMA). As control we used pLKO shRNA scramble expression vectors. Cells were then transduced with lentiviral particles in the presence of polybrene (8 μg/mL, Sigma). The cells were seeded at a density of 30,000 cells per well in a 24-well plate and allowed to adhere overnight. The next day, the cells were infected with the lentiviral particles for 6 h. Stably transduced cells were obtained using puromycin resistance. The nucleotide sequences of shRNA are listed in Supplementary **Table S4.**

### Organoids growth and treatment

2000 colon CSCs were plated in a 25 µL drop of matrigel in 48-well flat bottom plates in presence of rNODAL recombinant protein (50 ng/mL), SB431542 (10 μM) or 5-fluorouracile (5-FU) (25 μg/mL). Media were changed every 2 days including fresh rNODAL, SB431542 or 5-FU. At the indicated time points the organoids were counted in order to follow the growth of seeded cells. Counting was performed using ImageJ software. Triplicate wells were assayed for each condition and standard deviation (s.d.) was determined.

### Plasmid construct and transfection

NODAL expressing plasmids and empty vectors (pcDNA3.1) were obtained from Genscript (New Jersey, USA). Transfections were performed with Lipofectamine 2000 reagent (Invitrogen, Carlsbad, USA) following the manufacturers' instructions.

### Apoptosis assay

Attached and floating cells were collected, resuspended and stained with Annexin V (550474; BD Bioscience) after incubation with Annexin V binding buffer (556454, BD PharMingen). Cells were then incubated with PI. Samples were analysed by flow cytometry using a FACS Canto II (BD), and data were analysed using DIVA Software.

### Cell cycle assay

To synchronize the cell cultures, the cells were seeded in 6-well plate in growth medium with 10% FBS overnight. Then the cultures were rinsed by PBS and changed to serum free medium. After serum starvation for 24 h, the cells were passaged and released into cell cycle by addition of serum. For FACS analysis, cell samples were harvested at indicated time points. Cells were trypsinised, washed in PBS, centrifuged, and pellets were fixed in 200 µL of 70% ethanol and stored at -20 °C until use. Cells were centrifuged and pellets resuspended in 200 µl of PBS with 10 µg/mL of RNAse A. Cells were incubated for 1 hat 37 °C prior to resuspension in PI. Cell-cycle analysis was carried out by flow cytometry (CANTO II). Data were analysed by DIVA software.

### Vitality assay

To assess organoids and cell lines viability, CCK8 assay was performed. Organoids and cells were incubated in the presence or absence of 25 μg/mL of 5-FU or pre-treated with 10 μM parthenolide (PDT) following 5-FU exposure. At the indicated time points cell vitality was measured.

### Xenotransplantation experiments

All experiments with mouse models were approved by the animal care and use committee of the Barcelona Science Park (CEEA-PCB), the Catalan Government (P18-R5-09) and by the local ministry (IACUC protocol #992/2017-PR).

Cells were injected subcutaneously, intra-caecum or intra-splenically in 5 to 6 weeks old NSG mice (Jackson Labs), which were followed until sacrifice. Tumor appearance was assessed by palpation.

### *In vivo* treatment of established colorectal cancer

Single-cell suspensions were subcutaneously injected and when the tumors reached 20 mm^2^ the mice were randomized to the respective treatment groups. Size and weight of the colorectal tumors were monitored. 5-FU was administered twice a week for 21 days (30 mg/kg i.p.). SB431542 was used at 25 mg/kg, by oral gavages twice daily for 4 weeks. Control mice were treated with vehicle.

### Statistical analyses

Results for continuous variables are presented as means ± standard deviation (SD) unless stated otherwise (n > 3). Treatment groups were compared to the independent samples t test. Pair-wise multiple comparisons were performed with the one-way ANOVA (two-sided) with Bonferroni adjustment. The disease-free interval of patients was calculated using the Kaplan-Meier method, and differences among subgroups were assessed by the log-rank test. Experiments were performed a minimum of three independent times and always performed in independent triplicate samples. qPCR were repeated a minimum of three independent times in triplicate. p < 0.05 was considered statistically significant. All analyses were performed using GraphPAD Prism7. Correlation analyses were performed applying the Pearson's correlation coefficient.

## Results

### Clinical impact of L1CAM, CXCR4 and Nodal signaling in CRC

**Figure 1:**
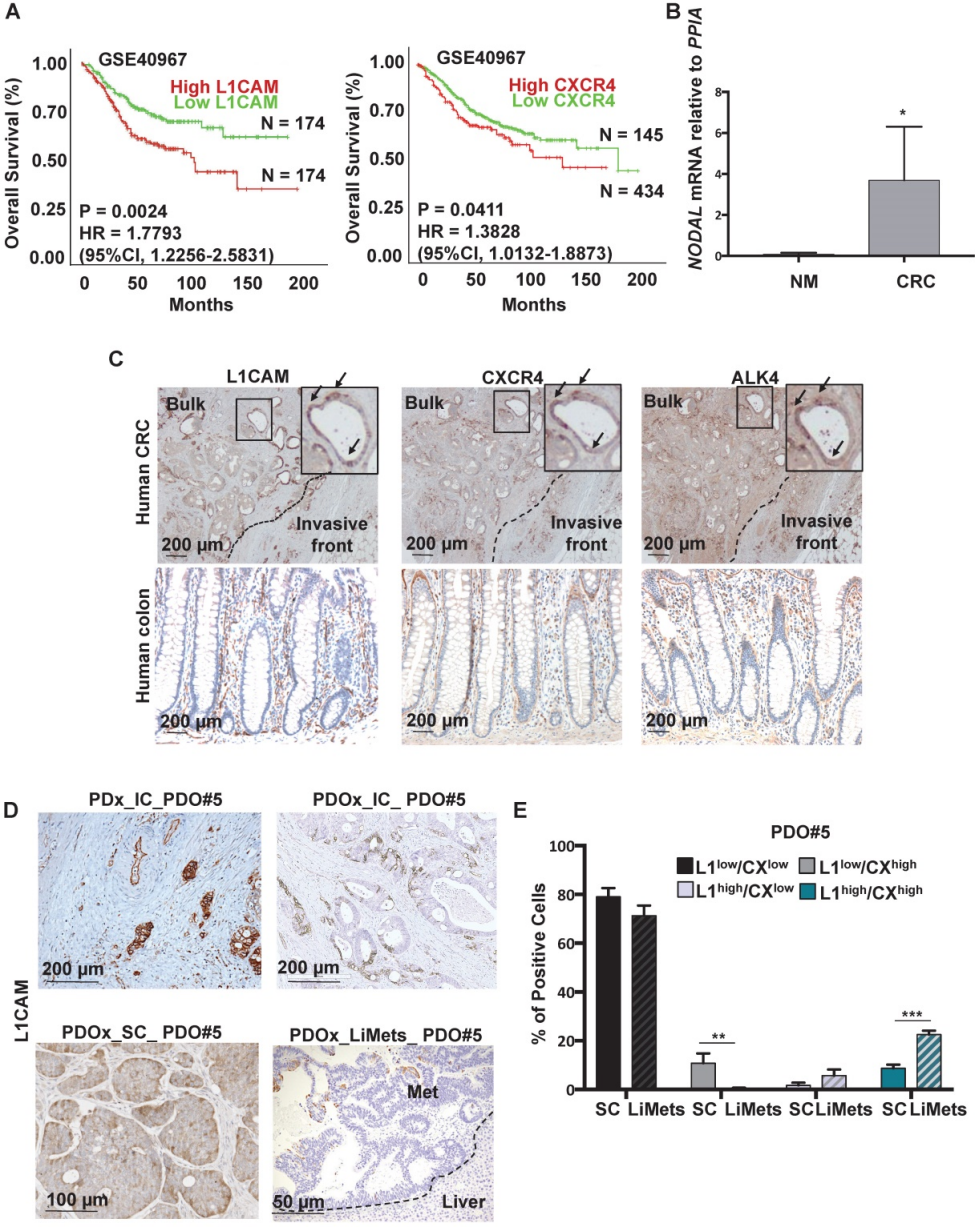
** Detection of L1CAM/CXCR4 subpopulation in human CRC.** (**A**) Kaplan-Meier curves showing overall survival of CRC patients, stratified according to the median value of *L1CAM* (n = 348, left panel) and *CXCR4* (n = 579, right panel) expression. A Median Group cut-off (50% High vs 50% Low) was used for L1CAM and (25% High vs 75% Low) was used for CXCR4. (**B**) qPCR analysis for *NODAL* in 3 Human Normal Mucosa samples and 10 Human CRC samples. Data are normalized to *PPIA* expression. *p<0.05. Data are mean ± SD. (**C**) Representative immunohistochemistry for L1CAM, CXCR4 and ALK4 (brown) in tissue sections from CRC patients (upper panel, n = 10) and normal human colon (lower panel, n = 3). (**D**) Representative immunohistochemistry for L1CAM (brown) of Patient-Derived-xenograft (PDx) generated from primary tumors injected in the caecum (IC) of immunocompromised mice, and their matched Patient-Derived-Organoids-xenograft injected in the caecum (PDOx_IC), subcutaneously (PDOx_SC) or their raised liver metastasis (LiMets). (**E**) L1CAM and CXCR4 staining % evaluated by flow cytometry in the PDOx_SC derived cells compared with the PDOx_LiMets derived cells. All cytometry gates were established based on isotype controls.**p<0.005, ***p<0.0005. Data are mean ± SD, n ≥6.

We first aimed to identify colorectal biomarkers with CSC features in tissue samples derived from patients with colorectal cancer. We observed that *L1CAM* and *CXCR4* overexpression was associated with reduced Overall Survival (OS) by Kaplan-Meier survival analysis (p < 0.05) (**Figure 1A**). Moreover a multi-gene analysis showed that patients with high expression of combined genes (i.e., *L1CAM*/*CXCR4*; *L1CAM*/*CXCR4/NODAL* and *L1CAM*/*CXCR4/ALK4)* have a reduced Overall Survival (OS) by Kaplan-Meier survival analysis (p < 0.05) (**Figure S1A-S1C**).

One of the key players in the (TGF)-β super family is NODAL, which has been detected at higher levels in CRC tissue compared to adjacent non-cancerous tissue 4. We analyzed by qPCR the level of *NODAL* in Human Normal Mucosa samples (n = 3) versus Human primary CRC (n = 10) and we observed a significant increase of *NODAL* expression in tumor samples as compared to healthy tissue samples (**Figure 1B**). The data were also confirmed in a series of CRC (T, n = 272) and adjacent normal colonic epithelium (N, n = 41) tissue samples from human patients (**Figure S1B**). Histological analyses on several primary tissues revealed that L1CAM, CXCR4 and ALK4 (the Nodal-receptor) have a similar expression pattern (**Figure 1C, insets**) and were anatomically localized in the bulk tumor and in the invasive front of colorectal cancer samples (**Figure 1C**). All samples from patients with colorectal cancer reproducibly demonstrated the presence of L1CAM, CXCR4 and ALK4 -positive cells in the invasive front with histological evidence for cell dissemination (**Figure 1C**). The high number of L1CAM^high^ and CXCR4^high^ cells in cancer tissue, compared to the normal epithelium (**Figure 1C**), most likely results from their oncogenic transformation.

To further investigate this phenotype we analyzed the expression of L1CAM in Patient-Derived-xenograft (PDx) generated from primary tumors injected in the caecum (IC) of immunocompromised mice, and their matched Patient-Derived-Organoids-xenograft injected either in the caecum (PDOx_IC), which were also able to give rise Liver Metastasis (LiMets) (**Figure 1D**), or subcutaneously (PDOx_SC) (**Figure S2A**). The histology profile of L1CAM was similar in all the sections analyzed (e.g., PDx_IC,PDOx_IC and PDOx_SC), with PDOx typically showing a more cuboidal appearance, but otherwise a comparable cellular morphology to the primary specimen (**Figure 1D**). Quantification of KI67 positive cells showed that L1CAM^high^ cells have a similar proliferation profile in the human CRC and in the PDOx_SC (**Figure S2B-C**). Interestingly, the L1CAM^high^ cells have a nuclear activation of pSMAD2 and are NODAL-positive in both human CRC and PDOx_SC (**Figure S2B**). Finally, also the CXCR4 expression profile is similar in both samples (**Figure S2B**). To more broadly assess the expression profiles of *L1CAM*, *CXCR4* and *NODAL* genes in CRC, we performed a quantitative PCR (qPCR) analysis on PDOx_SC, PDOx_IC, PDOx_LiMets (**Figure S2D**) and PDOs (**Figure S2E**). These results were consistent with the immunostaining and revealed similar expression levels among the different injections. Of note, by flow cytometry we found that L1CAM^high^/CXCR4^high^ population significantly increased in the PDOx_LiMets derived cells compared with the PDOx_SC derived cells (**Figure 1E**), suggesting that this double population plays a role in the secondary tumor.

### Nodal signaling and L1CAM^high^/CXCR4^high^ populations are both up-regulated in a hypoxic environment

One of the most frequently recognized features of tumor microenvironment is hypoxia, and hypoxic colorectal cancer cells seem to be poorly differentiated and express stem cell markers 16.

**Figure 2:**
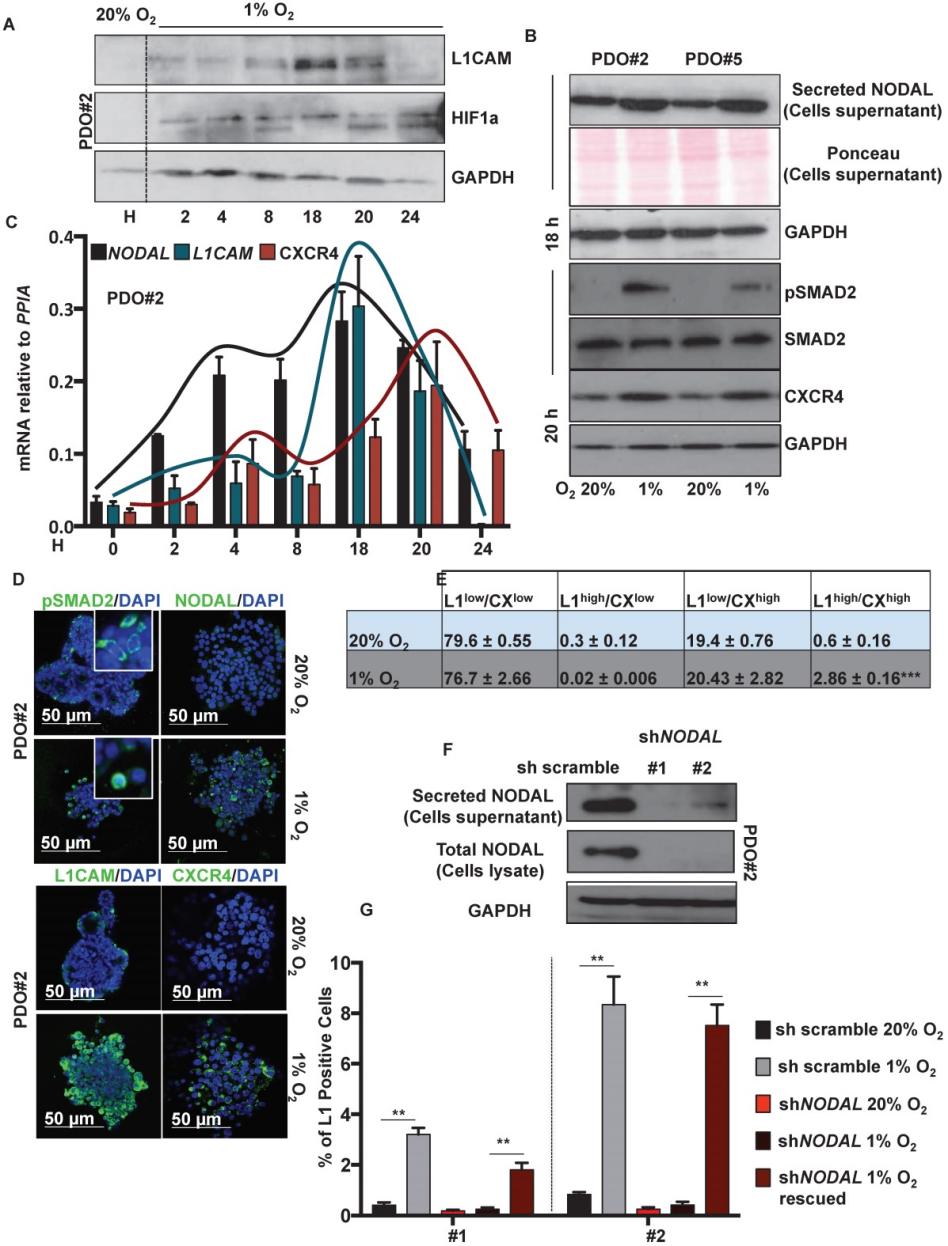
** Low levels of oxygen stimulates NODAL expression/secretion that in turns increases the L1CAM/CXCR4 population.** (**A**) Western blot analysis of L1CAM and HIF-1α in PDO#2 growth in normoxia (20% O_2_) or hypoxia (1% O_2_) at the indicated time. Parallel GAPDH immunoblotting was performed. (**B**) Western blot analysis of secreted NODAL, pSMAD2, SMAD2 and CXCR4 in PDO#2 and PDO#5, after 18 and 20 h respectively, in normoxia and hypoxia. Parallel Ponceau for the cells supernatant and GAPDH immunoblotting was performed. (**C**) qPCR analysis for *NODAL*, *L1CAM* and* CXCR4* in PDO#2 growth in normoxia and hypoxia at the indicated time. Data are normalized to *PPIA* expression. Data are mean ± SD, n ≥6. (**D**) Representative confocal images for pSMAD2 (the inset shows a magnification of the picture), NODAL, L1CAM, CXCR4 (green) and nuclei (blue, DAPI) of PDO#2 growth in normoxia or hypoxia for 18 h. (**E**) Flow cytometry quantification for L1CAM and CXCR4 in the PDO#2 growth in normoxia or hypoxia. All cytometry gates were established based on isotype controls. ***p<0.0005 compared with normoxia. N ≥3. (**F**) Western blot analysis of secreted NODAL and Total NODAL for PDO#2 clone #1 and clone #2. Parallel GAPDH immunoblotting was performed. (**G**) Flow cytometry quantification for L1CAM in the PDO#2 sh scramble or sh*NODAL*#1 and sh*NODAL*#2 growth in normoxia or hypoxia.The sh*NODAL*#1 and #2 growth in hypoxia were rescued with rNODAL. All cytometry gates were established based on isotype controls. **p<0.005 compared with sh*NODAL* in hypoxia. Data are mean ± SD, n ≥3.

Western blot analysis showed that L1CAM levels were up regulated, in a time-dependent manner, under hypoxic condition (i.e., 1% O_2_), with a peak of expression after 18 h (**Figure 2A**). NODAL and pSMAD2 similarly increased in the culture media after 18 h in hypoxic condition (**Figure 2B**, **upper panel**); while CXCR4 expression peaked later at 20 h (**Figure 2B, lower panel**). The expression profile of *NODAL*, *L1CAM* and *CXCR4* was confirmed by qPCR (**Figure 2C**) and immunofluorescence analysis (**Figure 2D**). Under low oxygen level we also detected, by immunofluorescence (IF) on PDO#2, a nuclear translocation of pSMAD2 (**Figure 2D**). Flow cytometry analysis confirmed that L1CAM^high^/CXCR4^high^ double population is significantly augmented in low oxygen compared to the normoxic condition (**Figure 2E** and **Figure S3A**). Notably, the increase of L1CAM population in low oxygen condition strictly relies on NODAL signaling. Indeed, *NODAL* knock down (**Figure 2F**) prevented increased expression of L1CAM in hypoxic condition, which is rescued when the cells were treated with rNODAL, as shown by FACS analysis (**Figure 2G** and**Figure S3B**). Conversely, CXCR4 expression was not affected upon NODAL downregulation (data not shown).

### Nodal induces an L1CAM^high^/CXCR4^high^ double population with CSC properties

**Figure 3:**
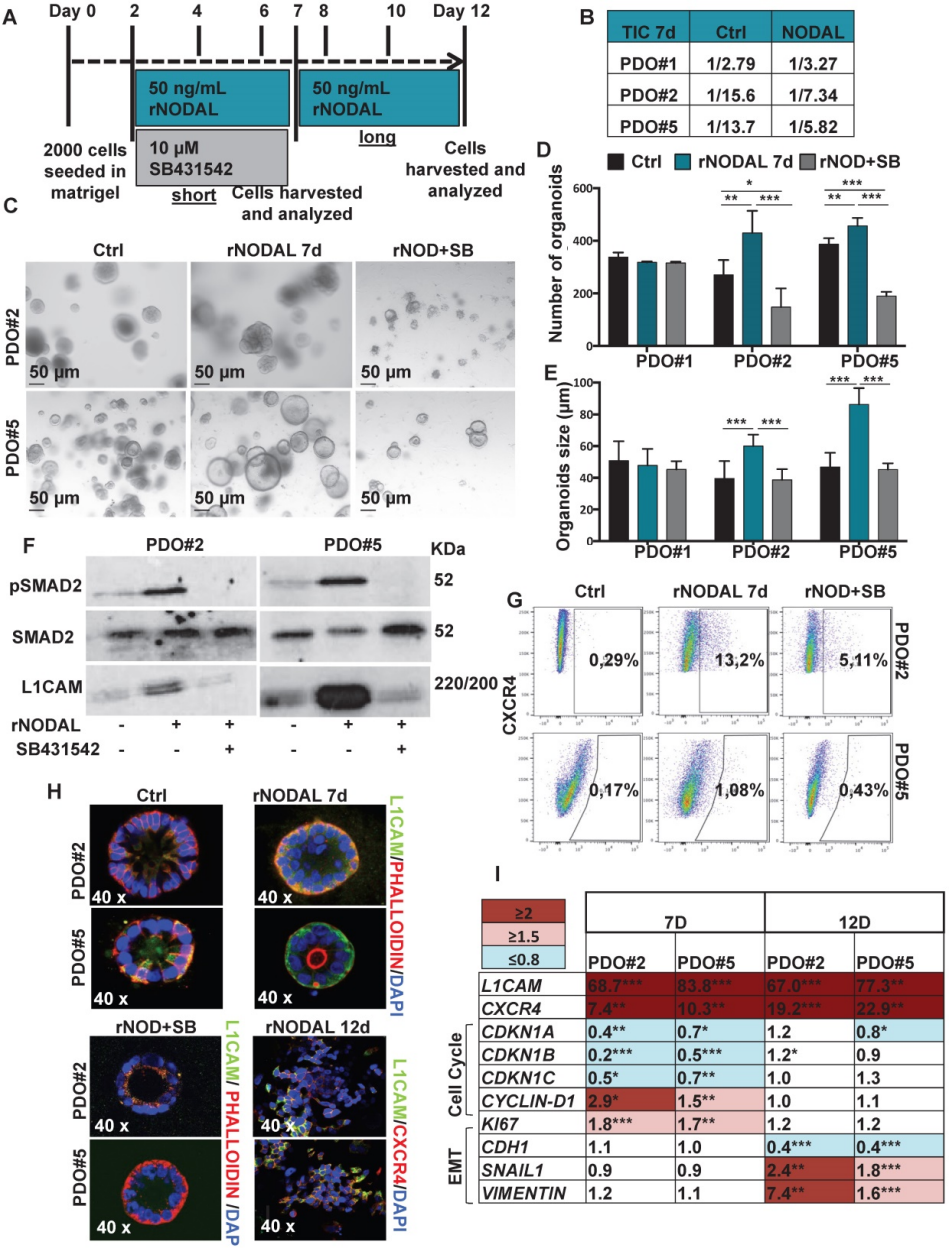
** Nodal induces L1CAM and CXCR4 expression in human CRC organoids.** (**A**) Schematic illustration of PDOs treatment: human organoids were treated with rNODAL alone or in combination with SB431542 for 7 day (short treatment) or 12 days (long treatment). After stimulation molecular analysis was performed. (**B**) Tumor initiation frequency (TIC) of PDO#1, PDO#2 and PDO#3 treated or untreated for 7 days with rNODAL. (**C**) Representative images of PDO#2 and PDO#5 treated or untreated with rNODAL for 7 days in presence or absence of SB431542. (**D**) Organoids formation capacity of PDO#1, PDO#2 and PDO#5 treated or untreated with rNODAL for 7 days in presence or absence of SB431542. *p<0.05, **p<0.005, ***p<0.0005 compared with control. Data are mean ± SD, n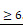 (**E**) Quantification of organoids size in PDO#1, PDO#2 and PDO#5 treated or untreated with rNODAL for 7 days in presence or absence of SB431542. ***p<0.0005 compared with control. Data are mean ± SD, n
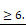
(**F**) Western blot analysis of pSMAD2, SMAD2, and L1CAM in PDO#2 and PDO#5 treated or untreated with rNODAL for 7 days in presence or absence of SB431542. (**G**) Representative flow cytometry analysis for CXCR4 in PDO#2 and PDO#5 treated or untreated with rNODAL for 7 days in presence or absence of SB431542. All cytometry gates were established based on isotype controls. N 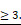 (**H**) Confocal images for L1CAM (green), phalloidin (red), CXCR4 (red) and nuclei (blue, DAPI) of PDO#2 and PDO#5 treated or untreated with rNODAL for 7 and 12 days in presence or absence of SB431542. (**I**) qPCR analysis of *L1CAM*, *CXCR4*, cell cycle and EMT genes in PDO#2 and PDO#5 treated or untreated for 7 and 12 day with rNODAL. Data are normalized to *PPIA* expressionand are presented as fold change in geneexpression relative tountreated cells. *p<0.05, **p<0.005, ***p<0.0005. n≥6.

High Nodal activity characterizes the colon cancer stem cell (CSC) population 4 and its expression is augmented in hypoxic condition as demonstrated above. To better understand the effect of Nodal on tumor cell behavior, we forced the system by treating the PDOs with NODAL recombinant protein (rNODAL). We performed either a short (7 days) or a long (12 days treatment for 7 days) with rNODAL in presence or absence of the inhibitor of the Nodal/Activin receptor ALK4 (SB431542); and a long treatment with rNODAL for 12 days (**Figure 3A**). We treated three different PDOs, PDO#1 (smad4 mutant, no responsive to the treatment) and two smad4 proficient PDOs (i.e., PDO#2 and PDO#5). While short NODAL treatment increased the tumor initiation frequency (TIC) in PDO#2 and PDO#5 of 2 and 2.3 times, respectively, it does not alter TIC in the PDO#1, as expected (**Figure 3B**). Moreover, the short NODAL treatment significantly increased the number of organoids (**Figure 3C**-**D**) and their size (**Figure 3E**), compared to the untreated control. Both L1CAM and CXCR4 expression was strongly induced by NODAL, in the PDO#2 and PDO#5, while it was repressed by pharmacological inhibition of Nodal signaling (**Figure 3F**-**G**). Western blot analysis of pSMAD2 confirmed that Nodal signaling was properly activated in PDO#2 and PDO#5 (**Figure 3F**), but not in the PDO#1 cells (**Figure S3C**). These findings were further confirmed by IF (**Figure 3H**) and qPCR (**Figure 3I** and**Figure S3D**). Moreover, both NODAL overexpressing PDO#2 and PDO#5 showed marked increase in *L1CAM* mRNA compared to the mock control PDOs (**Figure S3E**).

Of note, the short NODAL treatment reduced the cells death, while co-treatment with SB431542 inhibitor reverted this effect and decreased the percentage of live cells (**Figure S4A**-**B**). In line with these findings, while expression of the cell cycle inhibitors *CDKN1A, CDKN1B* and *CDKN1C* decreased, *CYCLIN-D1* and *KI67* increased upon short Nodal treatment (**Figure 3I**). Finally, FACS-based EDU incorporation assay showed increased S-phase (**Figure S4C**-**D**). It has been reported that Nodal promotes an aggressive phenotype in several types of cancers by inducing EMT 17,18. However, short Nodal treatment did not alter the expression of key EMT genes, including *CDH1*/E-cadherin, *SNAIL* and *VIMENTIN* (**Figure 3I**).

We then assessed the effect of long NODAL treatment (i.e., 12 days). Of note, we found that it induced morphological changes in the PDOs, which became flat and showed L1CAM^high^/CXCR4^high^ spreading cells (**Figure 3H** and **Figure S5A**). While long NODAL treatment induced both *LICAM* and *CXCR4* overexpression as observed with short NODAL (**Figure 3I**), it did not alter the expression of cell cycle (**Figure 3I**). Conversely, long NODAL treatment determined a significant decrease of *CDH1* and increase of *SNAIL1* and *VIMENTIN* (**Figure 3I** and **Figure S5B**), suggesting an induction of a mesenchymal phenotype. In order to evaluate the impact of the L1CAM expression on tumor growth, we knocked-down (KD) its expression in PDO#2 by shRNAs. *L1CAM* KD PDO#2 cells showed a reduced proliferative capacity compared to the sh scramble control (**Figure S5C**), and delayed tumor growth when subcutaneously injected in nude mice (**Figure S5D**). In agreement with these data, *L1CAM* knockdown significantly downregulated the expression levels of stem cell-related genes, including *EPHB2* and *OLFM4* (**Figure S5E**). Taken together these data supported the idea that Nodal signaling tightly control the cells behavior in a time-dependent manner.

Since both L1CAM and CXCR4 have been implicated in YAP activation in other cancers 19,20 and YAP is known to enforce CSC phenotype in CRC GEMM (genetically engineered mouse model)^21^,^22^ we looked at the relationship between L1CAM and CXCR4 expression with regards to YAP activation in CRC cells. We first assessed YAP cellular localization in SW480 and SW620 L1CAM^low^ *vs* L1CAM^high^ and CXCR4^low^ *vs* CXCR4^high^ sorted cells upon treatment with recombinant Nodal. Immunofluorescence analysis showed cytoplasmic localization of YAP in both untreated and Nodal treated cells, indicating that Nodal stimulation does not result in L1CAM and CXCR4 dependent YAP nuclear translocation (**Figure S6A**). We did not perform the same experiments under low oxygen condition sowe do not exclude that a hypoxic environment can modulate YAP in L1CAM or CXCR4 cells. In line with this idea, qPCR analysis of YAP target genes (i.e., *CTGF*, *CYR61*, *ANKRD1* and *ITGB2*) in the aforementioned-sorted populations were not induced by Nodal in SW480, SW620 and PDO#2 cells. Considering the overall results we did not observe a dependence of YAP target on L1CAM and CXCR4 (**Figure S6B**).

### Identification of migrating L1CAM^high^/CXCR4^high^ subpopulation in CRC organoids

**Figure 4:**
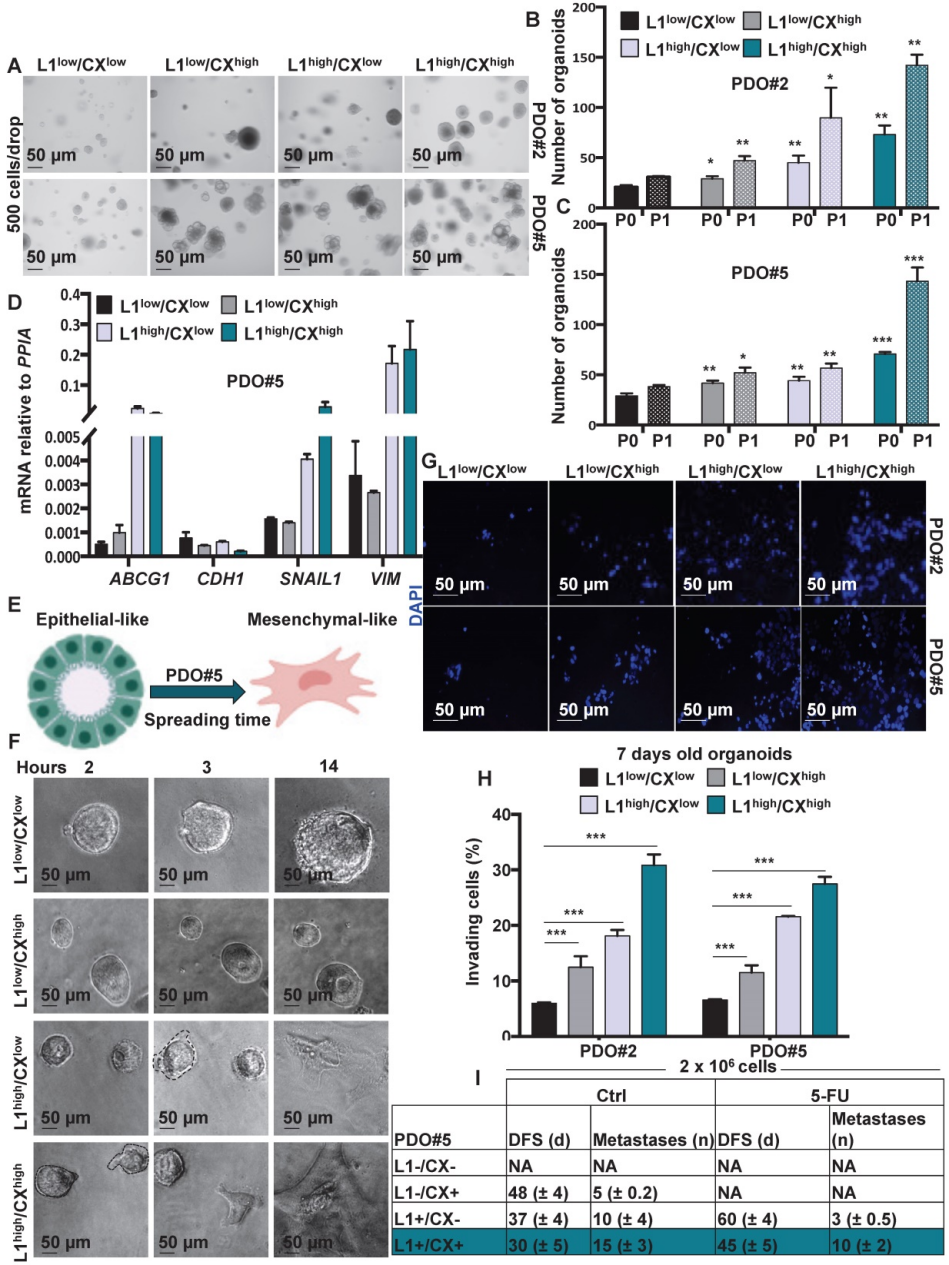
** Identification of migrating L1CAM^high^/CXCR4^high^ subpopulation in CRC organoids.** (**A**) Representative images of organoids derived from the four-sorted populations from PDO#2 and PDO#5. (**B-C**) Organoids formation capacity of the four-sorted populations from PDO#2 and PDO#5. P0 (passage 0): 7-days old organoids; P1 (passage 1): cells derived from P0 regrowth as organoids for additional 7 days. *p<0.05, **p<0.005, ***p<0.0005 compared with double low population. Data are mean ± SD, n≥6. (**D**) qPCR analysis for *ABCG1, CDH1, SNAIL1* and* VIM* in the four sorted populations from PDO#5. Data are normalised to *PP1A* expression. Data are mean ± SD, n≥6. (**E**) Representative scheme of PDO spreading from epithelial-like to mesenchymal-like phenotype. (**F**) Representative images of the four-sorted populations from PDO#5 after 2, 3 and 14 h after plating. (**G**) Representative images of organoids invasion ability (boyden chamber assay) of the four sorted indicated populations from PDO#2 and PDO#5. (**H**) Invasive potential of the four sorted indicated populations from PDO#2 and PDO#5. ***p<0.0005. Data are mean ± SD, n≥6. (**I**) *In vivo*disease free survival (DFS) and number of metastasis of intrasplenically injected four sorted indicated populations from PDO#5. Data are mean ± SD, n ≥ 6.

Since both L1CAM and CXCR4 have been associatedwith the migratory capacity of metastatic cells we decided to better understand the biology of L1CAM^high^/CXCR4^high^ double population. To this end, we FACS-sorted the cells from PDO#2 and PDO#5 and analyzed their capacity to form organoids after 7 days in culture (P0) (**Figure 4A** and**Figure S7A**). The L1CAM^high^/CXCR4^high^ cells exhibited the highest capacity to form organoids (**Figure 4B-C**) that were also bigger in size comparedto the other populations (i.e., L1CAM^low^/CXCR4^low^, L1CAM^high^/CXCR4^low^ and L1CAM^low^/CXCR4^high^) (**Figure S7B**). These results were further confirmed in the second generation (P1, the 7-days old organoids were enzymatically dissociated and regrow for additional 7 days) (**Figure 4B-C**). Accordingly, we found that cells L1CAM^high^/CXCR4^high^ cells showed higher expression of colon CSC markers (i.e., *LGR5*, *OLFM4* and *EpHB2*) and lower expression for the epithelial differentiation-associated gene (i.e.,*KRT20* and *MUC2*) compared to the L1CAM^low^/CXCR4^low^ population (**Figure S7C**). Furthermore, L1CAM^high^/CXCR4^low^ and L1CAM^high^/CXCR4^high^ populations also showed increased expression of the chemoresistant gene *ABCG1* and mesenchymal genes (i.e., *SNAIL1*and *VIMENTIN*), while expression of the epithelial gene *CDH1* was downregulated (**Figure 4D** and**Figure S7D**), suggesting that thesepopulations were more resistant to chemotherapeutic treatment and showed a more mesenchymal traits. Corroborating these data, time lapse indicated that L1CAM^high^/CXCR4^low^ and L1CAM^high^/CXCR4^high^ populations showed visible capacity to spread and move through the Matrigel (**Figure 4E**-**F** and**Figure S7E**). Moreover, the L1CAM^high^/CXCR4^high^ population started to spread earlier (i.e., 2 h after plating) than the L1CAM^high^/CXCR4^low^ (i.e., 3 h after plating). We never observed spreading of the L1CAM^low^/CXCR4^low^ and L1CAM^low^/CXCR4^high^ population even after 24 h after plating (**Figure 4F**). The higher aggressiveness of L1CAM^high^/CXCR4^low^ and L1CAM^high^/CXCR4^high^ was also confirmed by Boyden chamber invasion assay (**Figure 4G-H**). Then, we evaluated the metastatic potential of isolated colorectal L1CAM^low^/CXCR4^low^, L1CAM^low^/CXCR4^high^, L1CAM^high^/CXCR4^low^ nd L1CAM^high^/CXCR4^high^ populations. More specifically, we intrasplenically injected the four populations and after 5 days the mice were treated with either vehicle or 5-FU (biweekly with 25 mg/kg by i.p. administration for 21 days). In both control (i.e., Ctrl, vehicle) and 5-FU injected mice we did not observe any metastasis in the mice injected with the L1CAM^low^/CXCR4^low^ population. On the contrary, we observed a sequential reduction in the disease free survival (DFS) and increase number of metastasis in the mice intrasplenically injected with: L1CAM^low^/CXCR4^high^, L1CAM^high^/CXCR4^low^ and L1CAM^high^/CXCR4^high^ populations (**Figure 4I**). When treated with 5-FU we did not observe any metastasis in the mice injected with L1CAM^low^/CXCR4^high^ population. Moreover, in the 5-FU treated mice we observed a delay in metastasis formation of 23 days and 15 days (DFS) in the L1CAM^high^/CXCR4^low^ and L1CAM^high^/CXCR4^high^, respectively, compared to the Ctrl cells. The number of metastasis was also reduced from 10 to 3 in the L1CAM^high^/CXCR4^low^ and from 28 to 20 in L1CAM^high/^CXCR4^high^. These data indicated that the chemotherapy eradicated completely the capacity of L1CAM^low^/CXCR4^high^ to give rise to metastasis while, albeit with a reduced incidence, the L1CAM^high^/CXCR4^low^ and L1CAM^high^/CXCR4^high^ were still able to give rise to metastasis with a progressive increase in aggressiveness.

### Nodal inhibition chemosensitizes L1CAM^high^/CXCR4^high^ to chemotherapy

**Figure 5:**
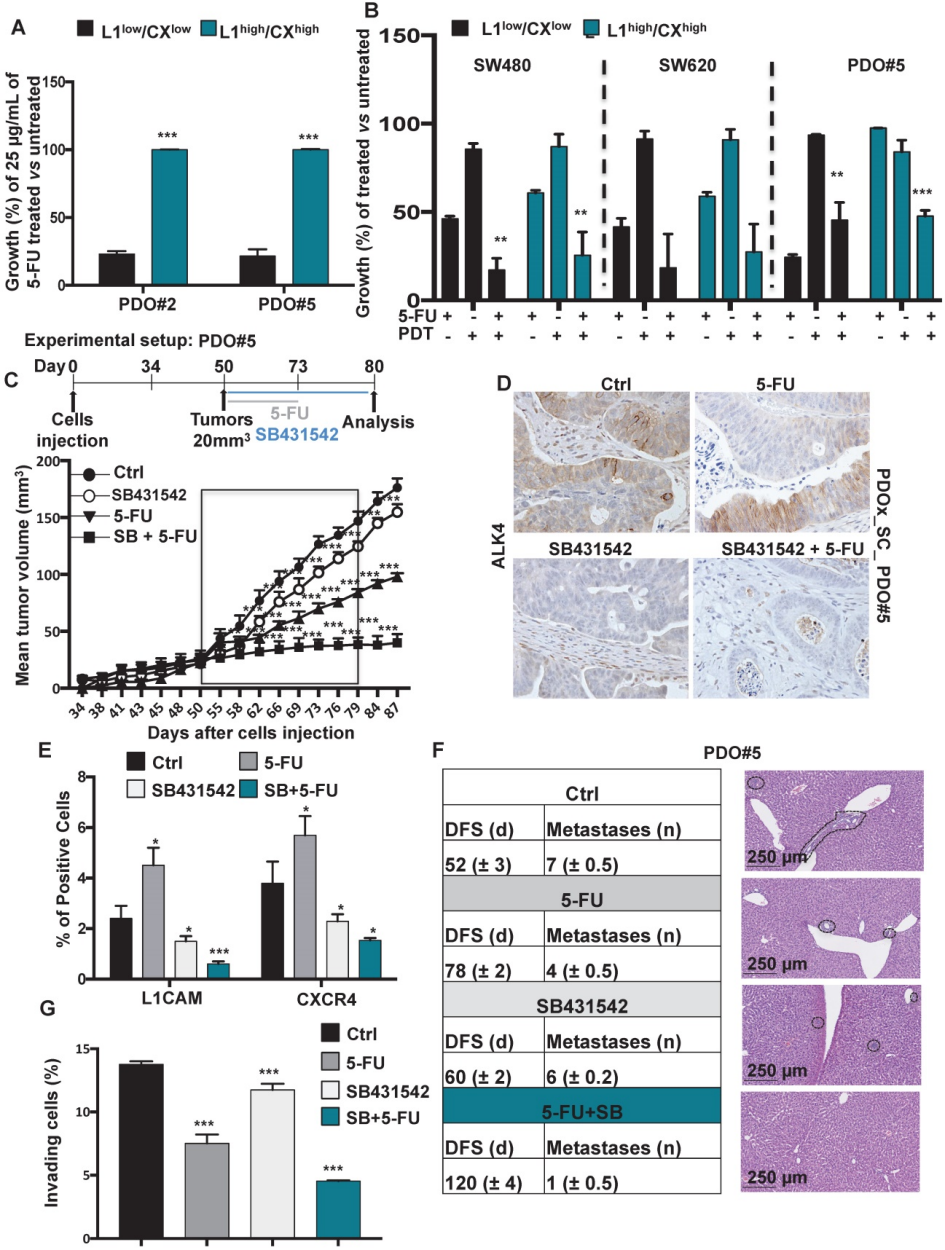
** Identification of chemoresistant L1CAM^high^/CXCR4^high^ subpopulation in CRC organoids.** (**A**) Growth capacity of the sorted indicated populations, from PDO#2 and PDO#5, in thepresence or absence of 5-FU. ***p<0.0005 compared to the low counterpart organoids. *N* ≥ 6. (**B**) Growth capacity of the sorted indicated in the presence or absence of 5-FU after pretreatment with PDT. ** p<0.005, ***p<0.0005 double treatment (5-FU + PDT) compared to the single treatment 5-FU *n* ≥ 6. (**C**) *In vivo* tumor growth of subcutaneously injected PDO#2 human organoids treated or untreated with 5-FU in the presence or absence of SB431542. Tumour size was measured every 2-5 days and tumor volume was calculated. Data are shown as mean (points) ± s.d. ***p<0.0005 compared to untreated mice. N = 8. (**D**) Representative immunohistochemistry for ALK4 in tissue sections from PDO#5_SC human organoids treated or untreated with 5-FU in the presence or absence SB431542. (**E**) Percentage of L1CAM and CXCR4 positive cells evaluated by flow cytometry in the tumor generated from PDO#5 organoids treated or untreated with 5-FU in the presence or absence of SB431542. All cytometry gates were established based on isotype controls. *p<0.05, ***p<0.0005. n≥6. (**F**) *In vivo* disease free survival (DFS) and number of metastasis of intrasplenically injected PDO#5 cells. Data are mean ± SD, n ≥ 6. (**G**) Invasive potential of the PDO#5 treated with 5-FU, SB431542 or SB + 5-FU compared to untreated cells. ***p<0.0005. Data are mean ± SD, n ≥6.

Colorectal cancers are fundamentally resilient to treatment because the malignant cells survive to the chemotherapy. Because CSC may play a crucial role in treatment resistance, we performed vitality analyses (i.e., CCK8 assay) of both L1CAM^low^/CXCR4^low^ and L1CAM^high^/CXCR4^high^ cells after exposure to the standard chemotherapeutic agent 5-FU. We found that L1CAM^high^/CXCR4^high^ cells showed dramatic drug resistance to 5-FU compared to autologous L1CAM^low^/CXCR4^low^ cells (**Figures 5A**). Both CXCR4 and L1CAM have been implicated in the NFkB activation that can in turn cause chemoresistance and thus contribute to the colorectal CSC phenotype 23,24. To determine whether NFkB inhibition can prevent chemoresistance in the L1CAM^high^/CXCR4^high^ compared to the low counterpart cells we pretreated them with non-toxic dose, i.e. 10 μM, of the NFkB inhibitor Parthenolide 25 for 24 h. Then, we treated the cells with 25 μg/mL of 5-FU (every 2 days for 4 days) and analyzed the chemoresistance by CCK8 vitality assay at day 7. Interestingly, we observed that NFkB inhibition indeed prevented the chemoresistance in the L1CAM^high^/CXCR4^high^ cells compared to L1CAM^low^/CXCR4^low^cells (**Figure 5B**).

To demonstrate the *in vivo* relevance of these findings, we analyzed tumor samples from mice bearing colorectal cancer after injection of 1×10^6^ PDO#5 cells and receiving either vehicle or 5-FU treatment (biweekly with 25 mg/kg by i.p. administration for 21 days) when tumors reached 20mm^3^. Based on our idea that Nodal signaling regulates the aggressiveness of the tumor cells through L1CAM and CXCR4, we investigated whether inhibition of Nodal signaling by SB431542 translates into increased progression-free survival in pre-established colorectal cancers. Over time, both control and SB431542 treated animals bore large, life-limiting tumors and succumbed within 87 days after tumor implantation. 5-FU alone significantly prolonged survival due to inhibition of tumor growth, but all animals showed progressive disease. Interestingly, the combination of SB431542 and 5-FU significantly increased the long-term survival compared to 5-FU alone, with 100% survival at day 87 (**Figure 5C**).

Harvesting the tumors after the last round of SB431542 treatment administration revealed efficient *in vivo* targeting of the Nodal pathway (**Figure 5D**) with subsequent downregulation of L1CAM and CXCR4 (**Figure 5E**). Although the tumors in the 5-FU treated mice were significantly smaller compared to vehicle-treated mice (**Figure 5C**), a significant enrichment of L1CAM^high^ and CXCR4^high^ had occurred (**Figure 5E**). Excitingly, the double combination (i.e., SB431542 + 5-FU) almost completely eliminated the cells L1CAM^high^ and CXCR4^high^ cells (**Figure 5E**). Then, we evaluated the Nodal signaling inhibition both *in vivo* (**Figure 5F**) and *in vitro* (**Figure 5G**) in a metastatic setting. Specifically, mice were injected intrasplenically with 1×10^6^ PDO#5 cells and after 5 days they were treated with either vehicle (DMSO), 5-FU, SB431542 or a double combination of SB+5-FU. While the 5-FU injected mice showed a delay in metastasis formation and a reduction in the metastasis number compared to control, no significant differences were observed in SB431542 injected mice compared to vehicle. Interestingly, we observed a strong reduction in DFS and metastasis number when the mice were treated with both SB431542 and 5-FU (**Figure 5F**) pointing out for a synergistic effect of the two drugs. The reduced aggressiveness of the double treatment was also confirmed *in vitro* in a Boyden chamber invasion assay (**Figure 5G**).

Taken together, these data demonstrate that double therapy is capable of eliminating tumor-promoting colorectal CSCs *in vivo*, leading to long-term progression-free survival (**Graphical Abstract**).

## Discussion

L1CAM^high^ population has been recently defined asthe regenerative origin of metastasis-initiating cells in colorectal cancer 26. For further clinical insights, we reported that high expression of L1CAM, together with CXCR4, significantly correlated with poor overall survival in CRC patients. In addition, we also observed a higher expression of NODAL in tumor tissue compared to normal colonic epithelium. Remarkably, we found that Nodal receptor ALK4, L1CAM and CXCR4 were co-expressed in the same cells in patient'sbiopsies. Surprisingly, we found that those with the highest levels of NODAL and active NODAL signaling (pSMAD2 positive) had also very high L1CAM expression levels. Furthermore, tumor derived from PDOx_SC exhibited co-expression of L1CAM and NODAL or pSMAD2, suggesting again that L1CAM expression may be functionally linked to NODAL signaling. It is still unclear how intracellular or secreted L1CAM plays a critical role in stemness maintenance and tumor progression/metastasis of CSCs. One possible mechanism involves direct regulation of CSC stemness-related genes via activation of L1CAM by external NODAL signals. pSMAD2/3-activated-L1CAM may trigger stemness-related genes expression in downstream signaling pathways 11. Alternatively, the metastatic properties of secreted L1CAM may be responsible for its effects on cancer progression. Ganesh K*. et al*. recently reported that L1CAM interacts with metastatic genes involving the EMT process 10. It has been extensively reported that EMT is involved in the maintenance of colorectal CSC-like cells 27,28, suggesting that NODAL can similarly regulate L1CAM through disruption of E-CADHERIN and withdrawal of REST form L1CAM promoter. This mechanism might mediate stemness maintenance function of L1CAM. However, there is no evidence that NODAL signals activate the REST axis to regulate L1CAM/CSC function. Another mechanism by which L1CAM may affect CSCs, involves its secretion into the extracellular matrix. It may also be possible that secreted L1CAM triggers non-canonical TGF-β signaling via upregulation of ERK, and eventually promotes migration and invasion of CRC cells. The data on hypoxia and differential peaks of HIF1alpha and L1CAM expression are probably suggesting that HIF1 alpha is not the transcription factor occupying the promoter of L1CAM. A possible speculation is that HIF1 alpha directly regulates the expression of NODAL, which in turns will activate L1CAM expression through nuclear SMADs. In our study, we first demonstrate that the canonical NODAL signaling is directly linked to L1CAM expression and that L1CAM enhances the cell proliferation and differentiation in spheroidal culture, as well as it regulates the stemness of colon CSCs. L1CAM may be a potent colon CSC marker as well as a cellular contextual oncogene.

Here we report, for the first time to our knowledge, a correlation between L1CAM^high^/CXCR4^high^ and the NODAL signaling pathway in colon CSCs and CRC patients. Indeed, we found that, under low oxygen condition, NODAL is up regulated and positively regulates L1CAM and CXCR4 expression, thereby maintaining stemness. In CRC patients, hypoxic condition and elevated mRNA levels of NODAL are associated with poor outcome 29 and stemness 30, respectively. In our study, we focused on high NODAL-expressing cells, L1CAM^high^/CXCR4^high^ population in colon CSCs, and the role of L1CAM^high^/CXCR4^high^ in the regulation of stemness in CRC cells.

Low oxygen levels induce the expression of the embryonic morphogen Nodal 31 and L1CAM has been showed to be regulated by SMAD2/SMAD4 complex in different systems 32. Low levels of oxygen induced secretion of NODAL in PDOs and regulate the expression of L1CAM and CXCR4 in a time dependent manner, suggesting a *consecution temporum* of NODAL expression followed by L1CAM and ending with CXCR4. This temporal expression indicates that the appearance of the metastatic population implies as a first step the NODAL secretion. Notably, NODAL KD prevents the increase of L1CAM levels under hypoxic condition, clearly showing that NODAL is requires for L1CAM activation. On the contrary, CXCR4 levels are not affected in the absence of NODAL and they still increase in low oxygen condition, indicating the absence of a linear correlation between NODAL and CXCR4 expression. To deep inside the mechanism, we observed that the exogenous treatment with recombinant NODAL increased L1CAM and CXCR4 level in CRC organoids in a time dependent manner, with different readout. The acute/short treatment (7 days) induces a proliferative phenotype, increases the number and the size of organoids and boosts the cell vitality. Moreover, spheroidal culture cells overexpressing NODAL exhibit enhanced L1CAM (but not CXCR4) expression and secretion, suggesting that L1CAM expression may be functionally linked to NODAL signaling. On the other hand, the long term treatment with rNODAL leads to the appearance of L1CAM^high^/CXCR4^high^ population with a less proliferative phenotype but significantly more invasive capacity.

Finally, *in vivo* co-treatment of implanted tumors with 5-FU and the Nodal inhibitor SB431542 significantly reduces tumor formation and stabilizes tumor size over time. These findings are also observed in pancreatic cancer 33 indicating that inhibiting NODAL signaling is a powerful strategy to eradicate or at least stabilize the disease to a chronic level. of particular relevance, the intrasplaenic injection of the different cell populations (i.,e, L1^low^/CX^low^, L1^low^/CX^high^, L1^high^/CX^high^) clearly indicate that the double negative population was unable to migrate and give rise metastasis. We thus speculate that a triple combination of 5-FU + SB43152 and a CXCR4 inhibitor (e.g., AMD070) or L1CAM inhibitor could be an effective therapeutic strategy to eradicate the tumor.

## Conclusions

In conclusion, a novel role of L1CAM in the regulation of colon CSCs, and subsequent regulation of the stemness and aggressive metastatic properties of colon CSCs was identified for the first time. Our study identified an important therapeutic target and raised the possibility that L1CAM^high^/CXCR4^high^ population-targeting drugs could be used to suppress CSC-related metastasis following conventional therapy.

## Supplementary Material

Supplementary figures and tables.

## Abbreviations

CRC: colorectal cancer
CSC: cancer stem cells
PDO: patients-derived-organoids
ALK4: activin receptor type-1B
CXCR4: Chemokine (C-X-C motif) receptor 4
DMEM: Dulbecco's Modified Eagle Medium
OS: Overall Survival
IC: intra-caecal
SC: subcutaneous
LiMtes: liver metastasis
5-FU: 5-Fluorouracile
GEMM: genetically engineered mouse model
PDT: parthenolide
