# Supplementary material for: Nodal-induced L1CAM/CXCR4 subpopulation sustains tumor growth and metastasis in colorectal cancer derived organoids
Source: Theranostics. 2021 Mar 31;11(12):5686–99. doi: 10.7150/thno.54027 (PMC8058729; doi:10.7150/thno.54027)
Supplement: Supplementary file 1 — Supplementary figures and tables. [file thnov11p5686s1.pdf]

Figure S1

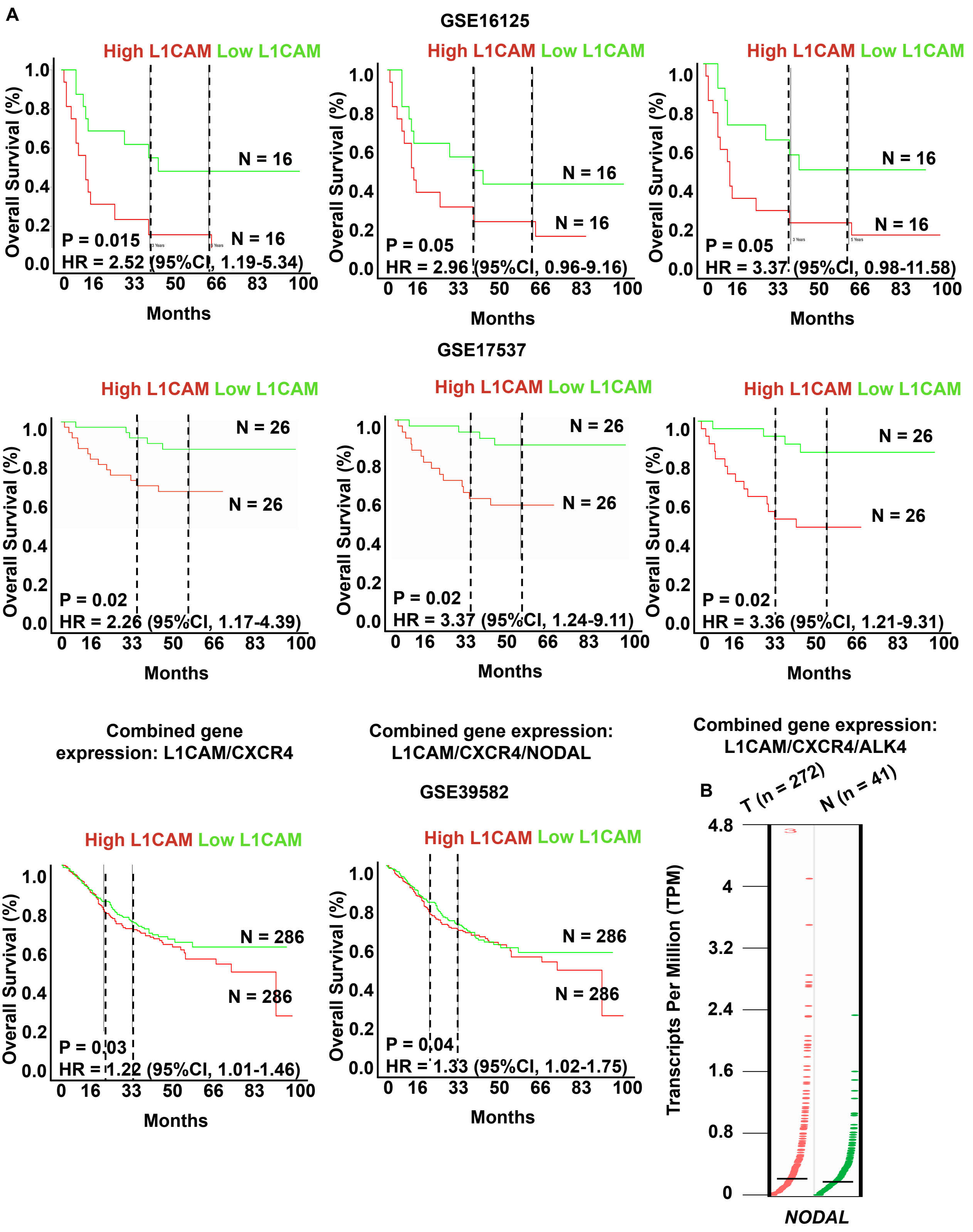
A
GSE16125
High L1CAM Low L1CAM
Overall Survival (%)
N = 16
N = 16
P = 0.015
HR = 2.52 (95%CI, 1.19-5.34)
Months
P = 0.05
HR = 2.96 (95%CI, 0.96-9.16)
P = 0.05
HR = 3.37 (95%CI, 0.98-11.58)
GSE17537
N = 26
N = 26
P = 0.02
HR = 2.26 (95%CI, 1.17-4.39)
P = 0.02
HR = 3.37 (95%CI, 1.24-9.11)
P = 0.02
HR = 3.36 (95%CI, 1.21-9.31)
Combined gene expression: L1CAM/CXCR4
Combined gene expression: L1CAM/CXCR4/NODAL
Combined gene expression: L1CAM/CXCR4/ALK4
GSE39582
N = 286
N = 286
P = 0.03
HR = 1.22 (95%CI, 1.01-1.46)
P = 0.04
HR = 1.33 (95%CI, 1.02-1.75)
B
T (n = 272)
N (n = 41)
Transcripts Per Million (TPM)
NODAL

Figure S1 - Detection of L1CAM/CXCR4 subpopulation in human CRC

(**A**) Kaplan-Meier curves showing overall survival of CRC patients, stratified according to the median value of *L1CAM* and*CXCR4* expression. A Median Group cut-off (50% High vs 50% Low) was used for L1CAM and (25% High vs 75% Low) was used for CXCR4. (**B**) Expression levels of *L1CAM*/CXCR4/*ALK4* in a series of CRC (T, n = 272) and adjacent normal colon (N, n = 41) tissue samples using GEPIA (Gene Expression Profiling Interactive Analysis) webserver.

Supplementary Figure 2

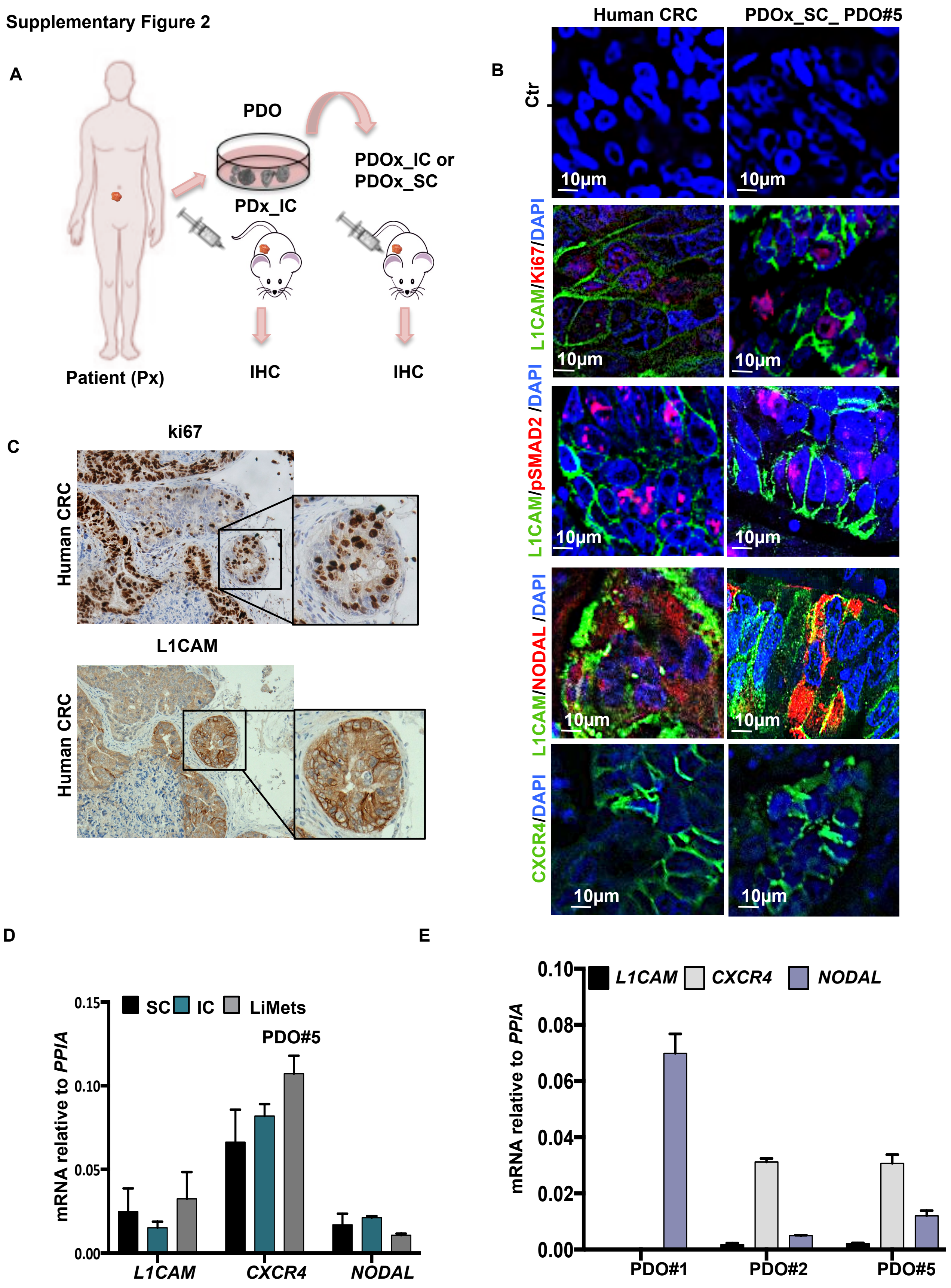

Figure S2 - Detection of L1CAM/CXCR4 subpopulation in human CRC

(**A**) Schematic representation of PDOs generation. PDx were isolated from fresh patient biopsies andinjected in the caecum (IC) of immunocompromised mice. Then, the cells obtained from enzymatic digestion of PDx_IC were cultured in matrigelas PDO and then subcutaneously (PDOx_SC) or intracaecum (PDOx_IC) injected in nude mice. (**B**) Confocal images for L1CAM (green), Ki67 (red), pSMAD2 (red), NODAL (red), CXCR4 (green) and nuclei (blue, DAPI) in the human CRC and PDOx-SC_PDO#5. (**C**) Representative immunohistochemistry forKi67 and L1CAM (brown) in tissue section from human CRC patients. (**D**) qPCR analysis of *L1CAM, CXCR4* and *NODAL* in PDO#5 human organoids. Data are normalized to *PPIA* expression. Data are mean ± SD, n ≥ *6.* (**E**) qPCR analysis for *L1CAM, CXCR4* and *NODAL* in PDO#1, PDO#2 and PDO#3. Data are normalized to *PPIA* expression. N ≥ 6.

Figure S3

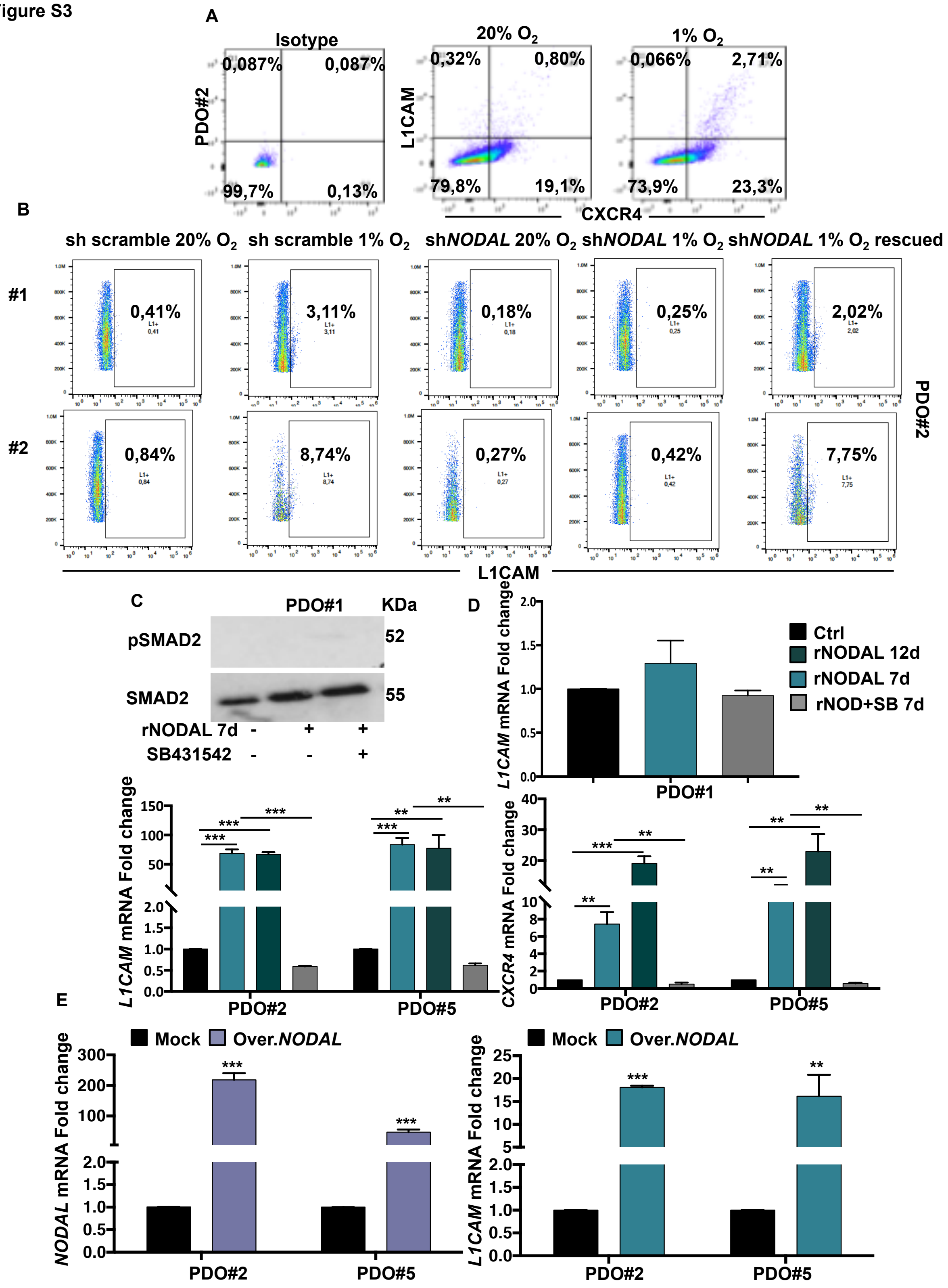
A
Isotype
20% O_2
1% O_2
PDO#2
L1CAM
CXCR4
0,087%
0,087%
99,7%
0,13%
0,32%
0,80%
79,8%
19,1%
0,066%
2,71%
73,9%
23,3%
B
sh scramble 20% O_2
sh scramble 1% O_2
shNODAL 20% O_2
shNODAL 1% O_2
shNODAL 1% O_2 rescued
#1
#2
0,41%
3,11%
0,18%
0,25%
2,02%
0,84%
8,74%
0,27%
0,42%
7,75%
PDO#2
L1CAM
C
PDO#1
KDa
pSMAD2
52
SMAD2
55
rNODAL 7d - + +
SB431542 - - +
D
L1CAM mRNA Fold change
Ctrl
rNODAL 12d
rNODAL 7d
rNOD+SB 7d
PDO#1
PDO#2
PDO#5
CXCR4 mRNA Fold change
E
Mock
Over.NODAL
NODAL mRNA Fold change
L1CAM mRNA Fold change

Figure S3 - Nodal induces L1CAM and CXCR4 expression in human CRC organoids

(**A**) Representative flow cytometry for L1CAM and CXCR4 in PDO#2 growth in normoxia or hypoxia. All cytometry gates were established based on isotype controls. N ≥ 3. (**B**) Representative flow cytometry analysis for L1CAM in PDO#2 sh scramble or sh*NODAL*#1 and #2 growth in normoxia or hypoxia.The sh*NODAL* #1 and #2 growth in hypoxia were rescued with rNODAL. N ≥ 3. (**C**) Western blot analysis of pSMAD2 in PDO#1 treated or untreated with rNODAL for 7 days in presence or absence of SB431542. Parallel SMAD2 immunoblotting was performed. (**D**) qPCR analysis of *L1CAM* and *CXCR4* in PDO#1, PDO#2 and PDO#5 treated or untreated with rNODAL for 7 and 12 days in presence or absence of SB431542. Data are normalised to *PPIA*expression and are presented as fold change (FC) in gene expression relative to control cells. **p<0.005, ***p<0.0005 compared with Ctrl. N ≥ 6.(**E**) qPCR analysis of *NODAL* and *L1CAM* in PDO#2 and PDO#5 transfected with control vecto r(mock) or *NODAL* overexpressing vector. Data are normalised to *PPIA* expression and are presented as fold change in gene expression relative to mock-transfected cells. **p<0.005, ***p<0.0005. n ≥ 6.

Figure S4

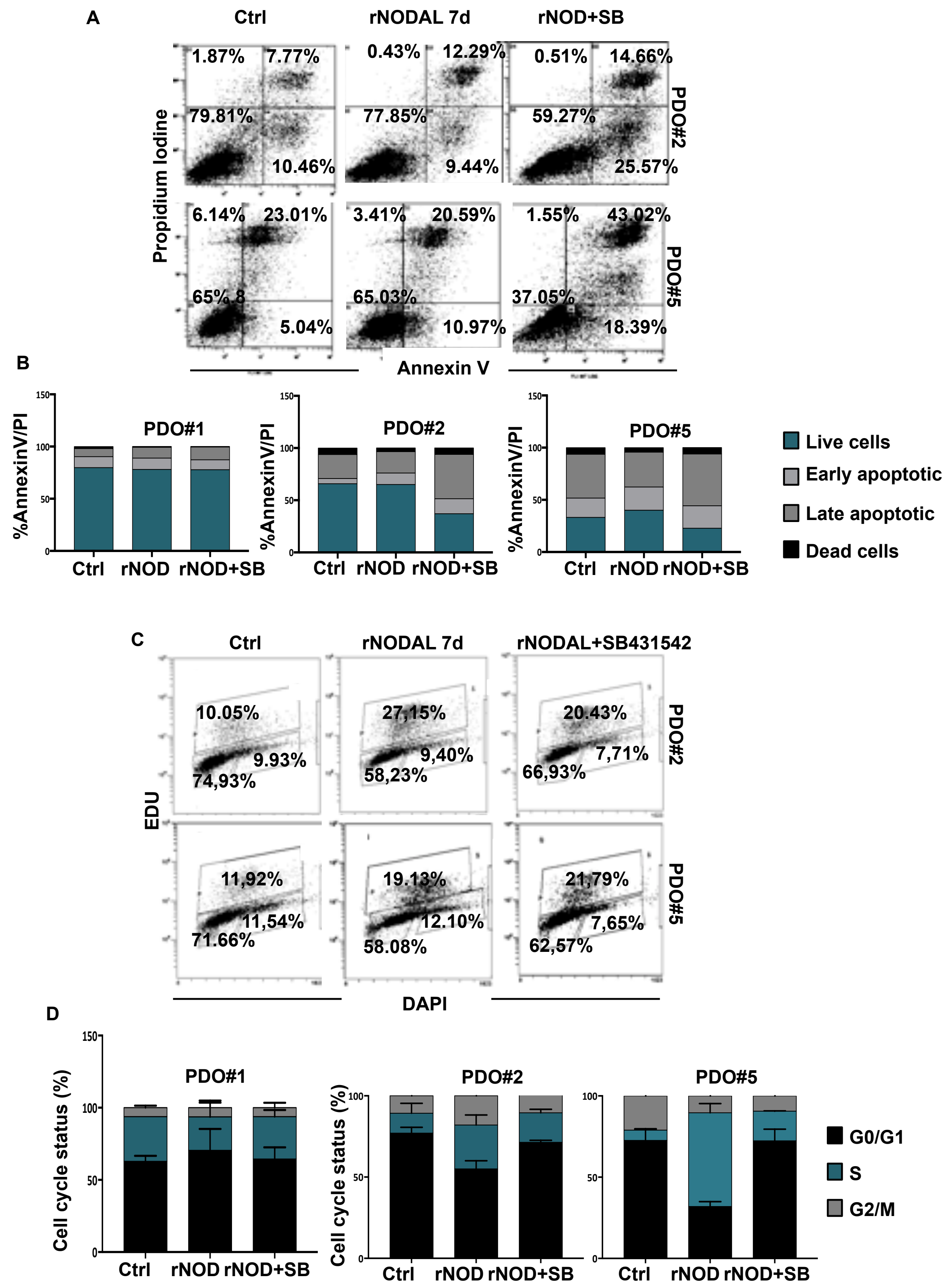
A
Ctrl
rNODAL 7d
rNOD+SB
Propidium Iodine
1.87%
7.77%
79.81%
10.46%
0.43%
12.29%
77.85%
9.44%
0.51%
14.66%
59.27%
25.57%
PDO#2
6.14%
23.01%
65%.8
5.04%
3.41%
20.59%
65.03%
10.97%
1.55%
43.02%
37.05%
18.39%
PDO#5
Annexin V
B
%AnnexinV/PI
PDO#1
PDO#2
PDO#5
Ctrl
rNOD
rNOD+SB
Live cells
Early apoptotic
Late apoptotic
Dead cells
C
Ctrl
rNODAL 7d
rNODAL+SB431542
EDU
10.05%
9.93%
74,93%
27,15%
9,40%
58,23%
20.43%
7,71%
66,93%
PDO#2
11,92%
11,54%
71.66%
19.13%
12.10%
58.08%
21,79%
7,65%
62,57%
PDO#5
DAPI
D
Cell cycle status (%)
PDO#1
PDO#2
PDO#5
Ctrl
rNOD
rNOD+SB
G0/G1
S
G2/M

Figure S4 - Nodal induces L1CAM and CXCR4 expression in human CRC organoids

(**A**) Representative flow cytometry plots for apoptotic cells as determined by AnnexinV/PI staining in PDO#2 and PDO#5 treated or untreated with rNODAL for 7 days in presence or absence of SB431542. (**B**) Percentage of apoptotic cells as determined by AnnexinV/PI staining in control PDO#1, PDO#2 and PDO#5 treated or untreated with rNODAL for 7 days in presence or absence of SB431542. (**C**) Representative flow cytometry plots for cell cycle analysis performed with EDU incorporationin PDO#2 and PDO#5 treated or untreated with rNODAL for 7 days in presence or absence of SB431542. (**D**) Percentage of PDO#1, PDO#2 and PDO#5 derived cellsin each phase of cell cycle treated or untreated with rNODAL for 7 days in presence or absence of SB431542.

Figure S5

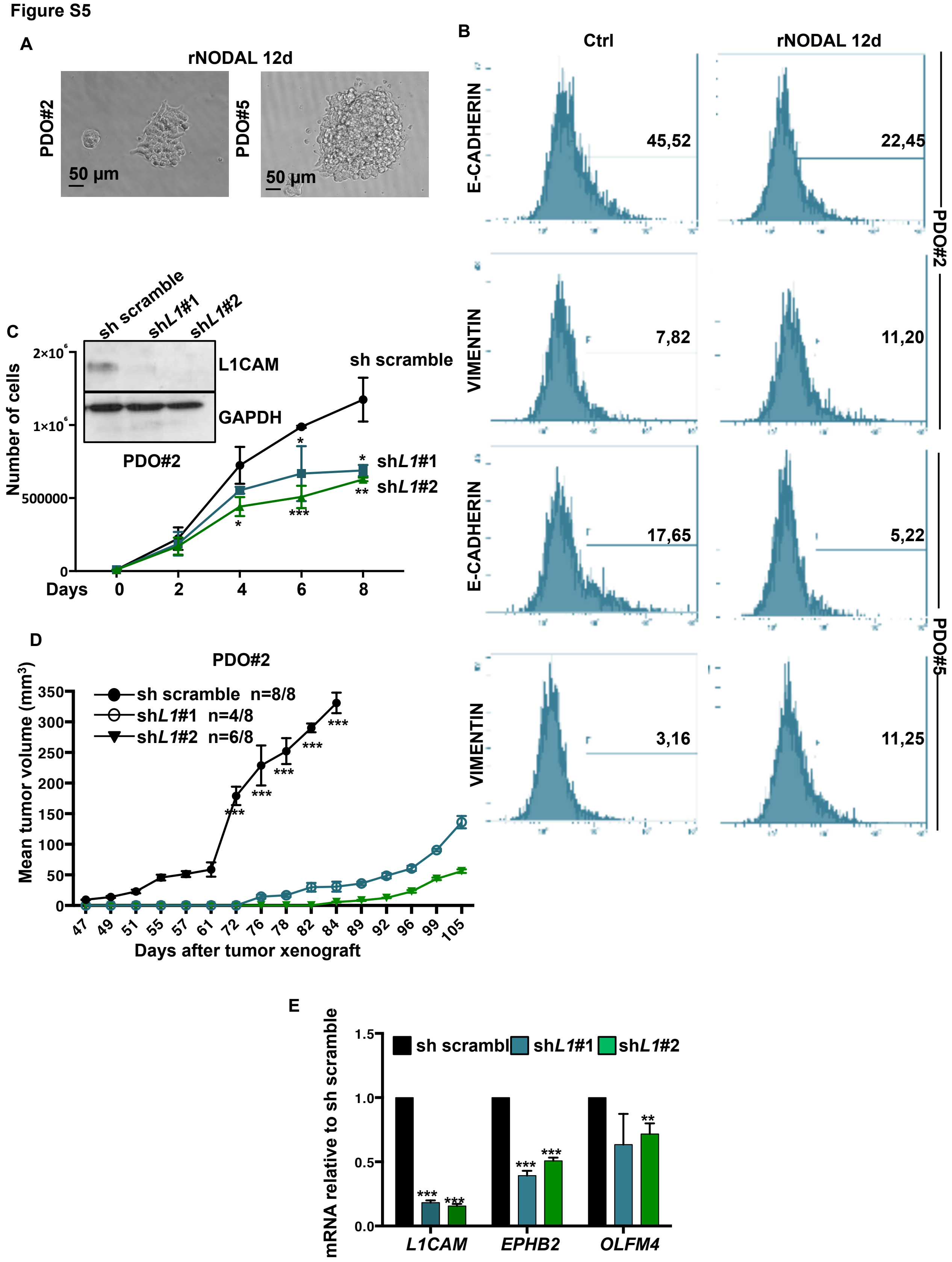

A
rNODAL 12d
PDO#2
PDO#5
50 µm
50 µm
B
Ctrl
rNODAL 12d
E-CADHERIN
45,52
22,45
VIMENTIN
7,82
11,20
PDO#2
E-CADHERIN
17,65
5,22
VIMENTIN
3,16
11,25
PDO#5
C
sh scramble
shL1#1
shL1#2
L1CAM
GAPDH
PDO#2
Number of cells
sh scramble
shL1#1
shL1#2
Days
D
PDO#2
Mean tumor volume (mm3)
sh scramble n=8/8
shL1#1 n=4/8
shL1#2 n=6/8
Days after tumor xenograft
E
mRNA relative to sh scramble
sh scrambl
shL1#1
shL1#2
L1CAM
EPHB2
OLFM4

Figure S5 - Identification of metastatic L1CAM^{high}/CXCR4^{high} subpopulation in CRC organoids

(**A**) Representative images of PDO#2 and PDO#5 treated with rNODAL for 12 days. (**B**) Representative flow cytometry plots of E-CADHERIN and VIMENTIN in Ctrl vs 12-days NODAL treated organoids. (**C**) Western blot analysis of L1CAM in PDO#2 sh scramble and sh*L1*. Parallel GAPDH immunoblotting was performed.Cells growth curves for PDO#2 sh scramble and sh*L1*. Each data point represents the mean ± SD of threeindependent experiments. **p < 0.005; ***p < 0.0005 compared to sh scramble. n ≥ 6. (**D**)*In vivo* tumor growth of subcutaneously injected PDO#2 control (sh scramble) and sh*L1*. Tumor size was measured every 2–5 days and tumor volume was calculated. Data are shown as mean (points) ± s.d. ***p<0.0005 compared to sh scramble. N = 8. (**E**) qPCR analysis for *L1CAM*, *EPHB2* and *OLFM4* in sh scramble and sh*L1* PDO#2 sh scramble and sh*L1*. Data are normalised to *PPIA* expression. **p<0.005 ***p<0.0005. n ≥ 6.

Figure S6

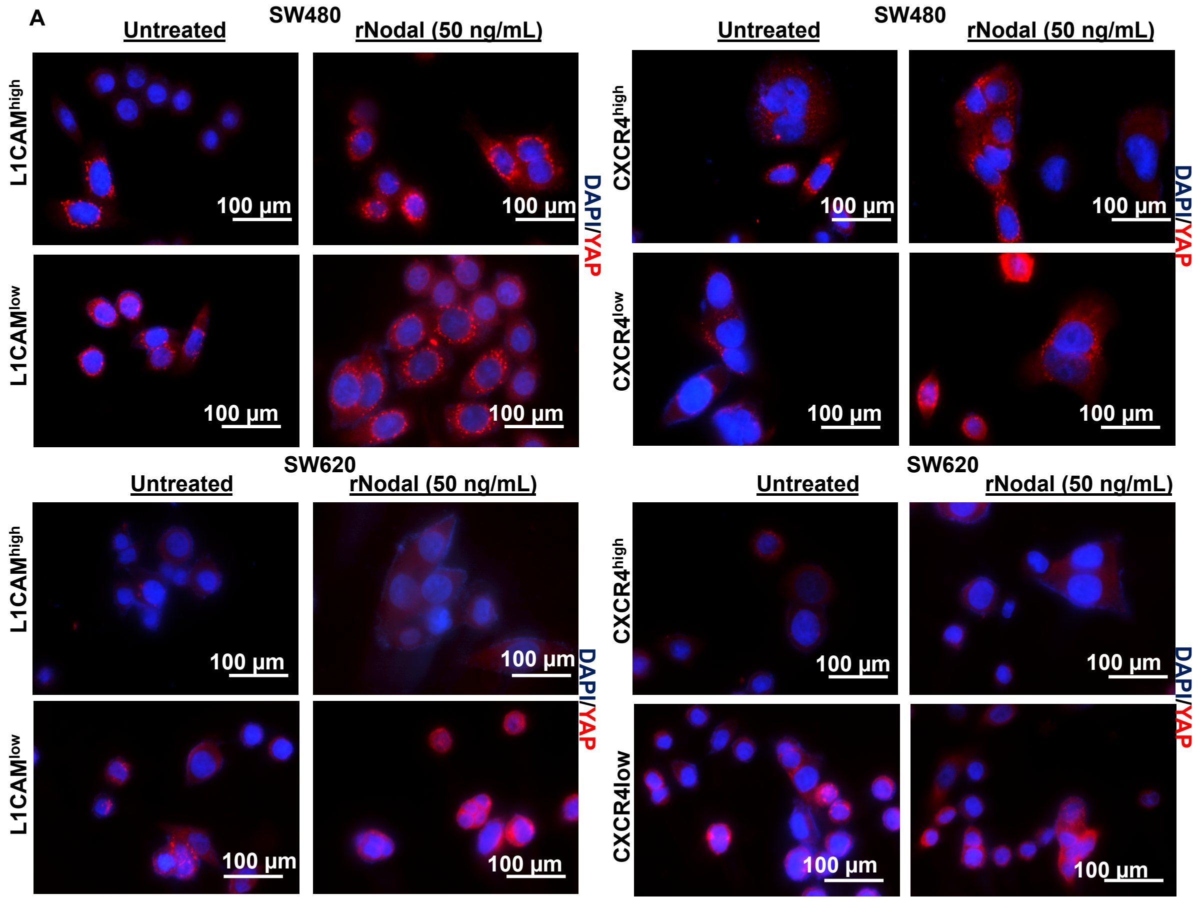

B

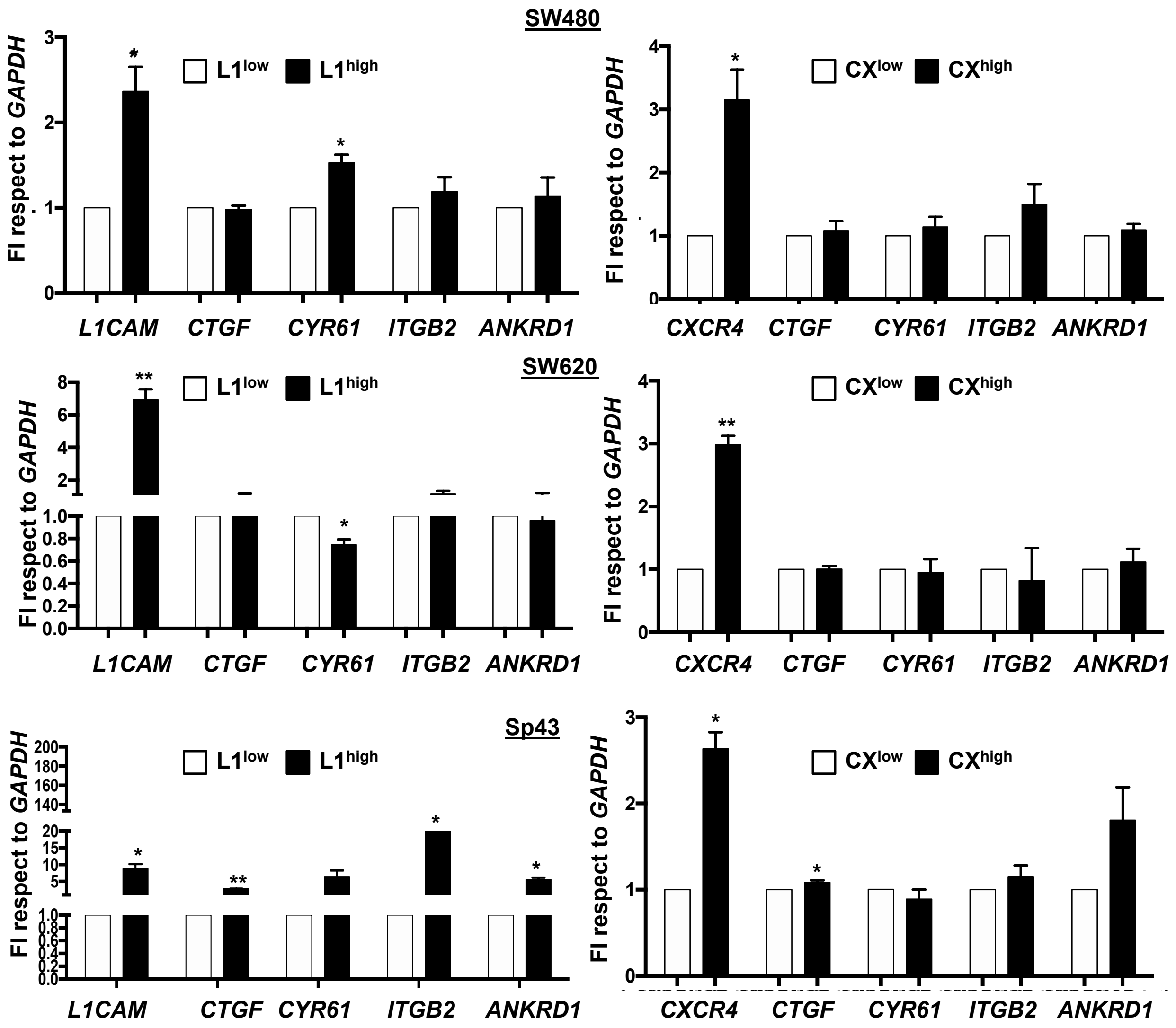

Figure S6 - Nodal stimulation does not cause an L1CAM and CXCR4 dependent YAP nuclear localization

(**A**) Confocal images for YAP (red) and nuclei (blue, DAPI) of SW480 and SW620 L1CAM or CXCR4 sorted cells treated or untreated with rNODAL (short treatment). (**B**) qPCR analysis of L1CAM, CXCR4, CTGF, CYR61, ANKRD1 and ITGB2 in SW480, SW620 and PDO#2sorted for L1CAM or CXCR4. Data are normalized to GAPDH expression and are presented as fold change in gene expression relative to untreated cells. *p<0.05, **p<0.005. n ≥ 6.

Figure S7

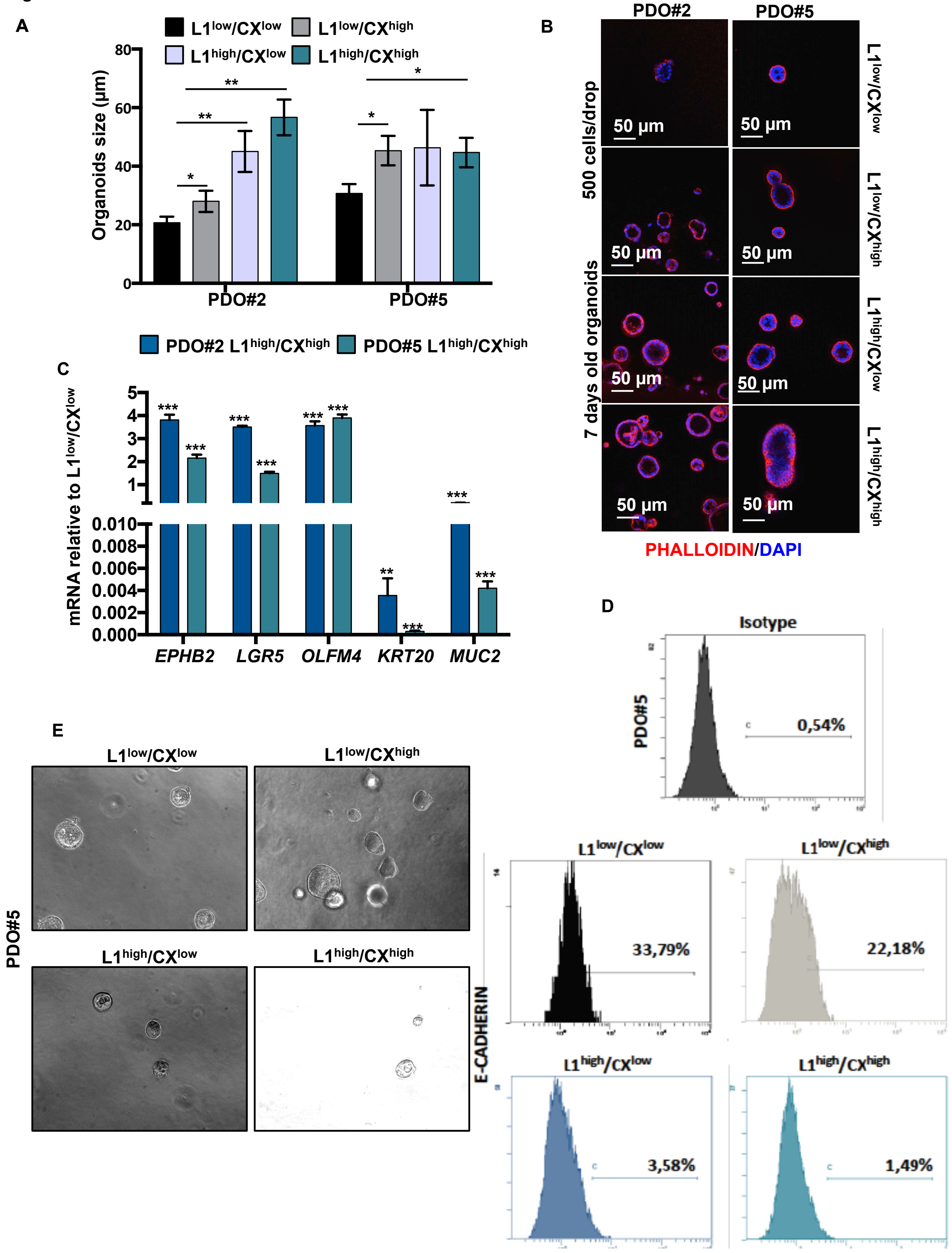
A
L1low/CXlow
L1low/CXhigh
L1high/CXlow
L1high/CXhigh
Organoids size (µm)
PDO#2
PDO#5
B
PDO#2
PDO#5
500 cells/drop
7 days old organoids
50 µm
PHALLOIDIN/DAPI
C
PDO#2 L1high/CXhigh
PDO#5 L1high/CXhigh
mRNA relative to L1low/CXlow
EPHB2
LGR5
OLFM4
KRT20
MUC2
D
Isotype
PDO#5
0,54%
E-CADHERIN
33,79%
22,18%
3,58%
1,49%
E
PDO#5

Figure S7 - Identification of metastatic L1CAM^{high}/CXCR4^{high}subpopulation in CRC organoids

(**A**) Quantification of organoids size in the four sorted indicated populations from PDO#2 and PDO#5. (**B**) Confocal images for phalloidin (red) and nuclei (blue, DAPI) of PDO#2 and PDO#5. (**C**) qPCR analysis for *EPHB2*, *LGR5*, *OLFM4*, *KRT20* and *MUC2*in the L1^{low}/CX^{low} and L1^{high}/CX^{high} populations sorted from PDO#2 and PDO#5. Data are normalised to *PPIA* expression. **$p<0.005$ ***$p<0.0005$. $n \geq 6$.(**D**) Representative flow cytometry plots of E-CADHERIN in the four sorted indicated populations. (**E**) Representative invasion assay videos of PDO#5 human organoids expressing different L1CAM and CXCR4 levels.

SUPPLEMENTARY METHODS

Antibody	Manufacturer and Reference	Technique and dilution used
Phospho-Smad2	Cell Signaling, Ref. 3108	Immunohistochemistry and Immunofluorescence (1/100), Western Blot (1/1000)
E-Cadherin-FITC	BD Bioscience Ref. 612130	Immunofluorescence (1/50)
Phalloidin-TRITC	Sigma Ref. P1951	Immunofluorescence (1/2500)
L1CAM-PE	eBioscience, Ref. 12-1719-42	Immunofluorescence and Flowcytometry (1/200)
Ki67-FITC	eBioscience, Ref. 11-5699-42	Immunofluorescence (1/200)
CXCR4-FITC	R&D systems, Ref. FAB170F-100	Immunofluorescence and Flowcytometry (1/100)
NODAL	Abcam, Ref:ab55676	Immunohistochemistry and Immunofluorescence (1/200)
YAP	Cell Signaling, Ref. 12395	Immunofluorescence (1/200)

TABLE S1- List of primary antibodies used.

Gene symbol	TaqMan Assay ID
ABCG1	Hs00245154_m1
CDH1	Hs01023894_m1
CDKN1A	Hs00355782_m1
CDKN1B	Hs01597588_m1
CDKN1C	Hs00175938_m1
CYCLIN-D1	Hs00765553_m1
CXCR4	Hs00237052_m1
EPHB2	Hs00362096_m1
KI67	Hs01032443_m1
L1CAM	Hs01109748_m1
NODAL	Hs00415443_m1
OLFM4	Hs00197437_m1
PPIA	Hs99999904_m1
SNAIL1	Hs00195591_m1
VIMENTIN	Hs00185584_m1

TABLE S2- List of TaqMan probes used for qPCR.

Gene symbol	Forwardprimer (5'->3')	Reverse primer (5'->3')
CYR61	AAACCCGGATTTGTGAGGT	GCTGCATTTCTTGCCCTTT
ANKRD1	AGACTCCTTCAGCCAACATGATG	CTCTCCATCTCTGAAATCCTCAGG
ITGB2	GATGAGAGCCGAGAGTGTGT	TCCTTCTCAAAGCGCCTGTA
YAP	TAGCCCTGCGTAGCCAGTTA	TCATGCTTAGTCCACTGTCTGT
CTGF	CTGCCTGGGAAATGCTGCGAGGAGT	GTTGGGTCTTGGGCCAAATGT
CXCR4	GGTGGTCTATGTTGGCGTCT	TGGAGTGTGACAGCTTGGAG
L1CAM	CACTATGGCCTTGTCTGGGA	ACATACTGTGGCGAAAGGGA
GAPDH	CAGGAGCGAGATCCCT	GGTGCTAAGCAGTTGGT

TABLE S3- List of primers used for qPCR (SYBR green method).

Symbol	OligoSeq
NODAL	CCGGGCGGTTTCAGATGGACCTATTCTCGAGAATAGGT CCATCTGAAACCGCTTTTTG
NODAL	CCGGGTGCTCCTAGATCACCATAAACTCGAGTTTATGGT GATCTAGGAGCACTTTTTG
L1CAM	CCGGCCACTTGTTTAAGGAGAGGATCTCGAGATCCTCTC CTTAAACAAGTGGTTTTTG
L1CAM	CCGGGCTAACCTGAAGGTTAAAGATCTCGAGATCTTTAA CCTTCAGGTTAGCTTTTTG

SCRAMBLE	CCGGCAACAAGATGAAGAGCACCAACTCGAGTTGGTGC TCTTCATCTTGTTGTTTTT

TABLE S4- List of nucleotide sequence of shRNA used.
